# Revisional Notes on the Cloud Forest Butterfly Genus *Oxeoschistus* Butler in Central America (Lepidoptera: Nymphalidae: Satyrinae)

**DOI:** 10.1007/s13744-019-00757-7

**Published:** 2020-03-14

**Authors:** T W Pyrcz, A Zubek, P Boyer, I Nakamura, B Wacławik, K Florczyk

**Affiliations:** 1 Nature Education Centre, Jagiellonian Univ., ul. Gronostajowa 5, 30-387 Kraków, Poland; 2 Entomology Dept., Institute of Zoology and Biomedical Research, Jagiellonian Univ, Kraków, Poland; 3 Le Puy Sainte Réparade, France; 4 New York, 14221 USA

**Keywords:** Costa Rica, female genitalia, *Oxeoschistus hilara lempira* n. ssp., *Oxeoschistus tauropolis mitsuko* n. ssp., Pronophilina, species diversity

## Abstract

So far, six species of *Oxeoschistus* Butler, including its junior synonym *Dioriste* Thieme, were listed from Central America, with five of them from Costa Rica alone, which appears to represent the highest regional diversity of this Neotropical montane butterfly genus. Our research based on field work, morphological studies and barcode analysis proved that one record is a misunderstanding perpetuated in scientific literature for over a century: *Oxeoschistus cothonides* Grose-Smith is identified here as an individual form of the female of *O. cothon* Salvin. The presence of *Oxeoschistus tauropolis* (Westwood) in Costa Rica, subject to some controversy, is confirmed, and a new local subspecies is described from Costa Rica, *O. tauropolis mitsuko* Pyrcz & Nakamura n. ssp. Specific status of *O. euriphyle* Butler is reinstated based on morphological and molecular data. A new subspecies *O. hilara lempira* Pyrcz n. ssp. is described from Honduras. *O. puerta submaculatus* Butler is reported for the first time from the Darién region on the Panama–Colombia border. Species relationships are preliminarily evaluated based on COI data concluding, among others, that *O. hilara* and *O. euriphyle* are less closely related than previously assumed. Altitudinal and distributional data are revised, and ecological and behavioural information of all the species of Central American *Oxeoschistus* is provided.

**Electronic supplementary material:**

The online version of this article (10.1007/s13744-019-00757-7) contains peer-reviewed but unedited supplementary material, which is available to authorized users.

## Introduction

Most groups of Neotropical montane Lepidoptera are heavily underrepresented in Central American mountains compared to the Andes (Pyrcz & Viloria 2012). Many species-rich genera in various families of diurnal and nocturnal Lepidoptera that contain dozens of Andean species are represented in Panama and Costa Rica, or further north in Guatemala and southern Mexico, by only one or two species. This pattern is particularly evident in the case of the satyrid subtribe Pronophilina (Nymphalidae, Satyrinae, Satyrini), one of the most diverse and species-rich taxa of cloud forest Neotropical butterflies with approximately 45 genera and well over 600 species (Lamas *et al*^2004^, Pyrcz 2010). This subtribe is represented in the mountains of Costa Rica, in the Meseta Central and the Cordillera de Talamanca that extends into Panama, by a modest 19 species (DeVries 1987), whereas in some Andean mountain ranges and countries the species richness is several orders of magnitudes higher (Pyrcz & Viloria 2012). This is not due to area effect as local counts along altitudinal gradients in some Andean localities give figures around or above 100 species (Pyrcz 2010). Most probably, such a low diversity of this and many other groups of butterflies in Central America reflects the history of radiation in montane butterflies, related to the uplift timing of the Andes and the recent availability of this region to south American colonizers with the formation of the Panama land bridge (Pyrcz & Viloria 2012, Pyrcz *et al*^2018^, Cassildé *et al*^2012^, De-Silva *et al*^2017^). At that time, the Andes, at least in the central part, had already reached their current elevations and were inhabited by rich faunas of butterflies (Casner & Pyrcz 2010). Even when the land connection was formed, a large gap in montane habitats of several hundreds of kilometres persisted between northern Andes and the recently united volcanic bridges in Central America. The most probable pathway for colonization of Costa Rican and Guatemalan mountains by southern elements was opened with the Pleistocenic Ice Ages in the process of slow range expansion thanks to the formation of cloud forest ecological corridors. Such corridors were apparently short lasting or not solid enough, and generally allowed the colonization of Central America only by montane species occurring in lower altitudinal strata. That is why the fauna of Costa Rican Pronophilina is represented by generally widespread species occurring in the Andes below 1600 m above the sea level or their allopatric relatives in such genera as *Pedaliodes* Butler 1867, *Praepronophila* Forster, 1964, *Lymanopoda* Westwood, 1851; and *Eretris* Thieme, 1905, two notable exceptions being *Drucina leonata* Butler, 1872, and *Arhuaco dryadina* (Schaus 1913) (Pyrcz *et al*^2018^). Furthermore, some widespread and rather diverse Andean genera with representatives occurring at lower elevations are absent in Central America including, for example *Corades* Hewitson, [1849]; *Lasiophila* C. Felder & R. Felder, 1859; and *Manerebia* Staudinger, 1897. Others, such as *Pedaliodes*, are extremely underrepresented, as the genus comprises well over 250 species in the Andes, but only seven in Central America (an overall proportion of 0.02).

The only exception to this otherwise general rule seems to be the genus *Oxeoschistus* Butler, 1862, including *Dioriste* Thieme 1907, sunk as a subjective junior synonym by Lamas *et al* (2004), an issue discussed somewhat more in detail by Pyrcz (2004). It comprises 14 currently recognized species, eight of which are distributed in the tropical Andes, one found in the Guyana Shield (*Oxeoschistus romeo* Pyrcz & Fratello 2005) and six reported from the mountains of Central America from Panama to south-central Mexico (Pyrcz 2010). This is a very high (0.42) proportion in the overall genus-level species richness, unparalleled among other genera of Pronophilina. In the Andes, even in the most species diverse areas such as the eastern slopes in Peru and southern Ecuador, the genus *Oxeoschistus* is represented, according to current systematic arrangement, by a maximum of four species in local faunas, distributed sympatrically and/or parapatrically along an altitudinal gradient. In Costa Rica, however, as many as five sympatric/parapatric species are reported, namely *Oxeoschistus cothon* Salvin, 1869; *Oxeoschistus cothonides* Grose-Smith, 1869; *Oxeoschistus euriphyle* Butler, 1872; *Oxeoschistus tauropolis* (Westwood, 1851) and *Oxeoschistus puerta submaculatus* Butler & Druce 1874, of which only the last species occurs outside Central America in the northern Andes.

Faunal data for Costa Rica are generally considered to be reliable, and Costa Rica is one of the countries, if not the country, where the butterfly fauna is best understood within the entire Neotropical region. Its history of explorations dates back to the second part of nineteenth century with the magnificent works of Godman and Salvin and culminated with the publication of DeVries’ field guides (DeVries 1987, DeVries 1997) which remain a prime source of information not only for Costa Rican butterflies but also for the rest of Central America. Since then, numerous important contributions were published over the years by Janzen and co-workers (Burns *et al*^2008^, Hebert *et al*^2004^), and another field guide by Chacón & Montero (2007). Nevertheless, the unexpectedly high species diversity in Costa Rica’s *Oxeoschistus* remains unexplained, and was therefore selected as one of the main topics in our research project on the distribution and taxonomy of Talamancan Pronophilina.

## Material and Methods

### Field studies

Field studies took place in Guatemala (TP, PB) in 2005, in Costa Rica in July–August 2015 (TP) and March 2016 (TP, PB), and independently from February–March, 2004, to May–June, 2013 (IN), in Panamá (IN), as well as in Honduras (Fernando Marabuto, Piotr Naks). The area sampled for Pronophilina and *Oxeoschistus* in Costa Rica was situated in the Cerro de la Muerte region in the western Cordillera de Talamanca, with transects established between La Georgina and División, División and Santa Eduviges, La Georgina and Cerro Buenavista in the Pacific Conservation Area. Volcanoes Turrialba and Irazú were also visited. The covered altitudinal range was from 1800 to 3400 m. Collecting was performed with standard entomological nets with extensions ranging from 1.5 to 2.5 m. Also, van Someren-Rydon traps were used. They were placed in the canopy at some 5–10 m and baited with rotten bananas. Additionally, bait consisting of dung and rotten fruits was placed on the ground along trails. A total of 25 working days were carried out by two people. IN’s work covered wider areas, including lower altitudes and at different times of the year. Additional distribution data were retrieved from the following websites: http://creativecommons.org/licenses/by/4.0/, www.inbio.ac.cr and www.butterfliesofamerica.com (Warren *et al* 2013). Distribution maps were produced with the use of Corel DRAW X3.

### Morphology

Male and female genitalia were dissected with the following procedure: abdomens were soaked in 10% KOH solution for 5–10 min at boiling temperature, and then cleaned out of soft tissue in water in order to better visualize soft tissue. Female abdomens were stained in chlorazol black in order to identify soft genital parts. Dissected genitalia were cleaned out of water by using ethanol 90% and 95% solutions. Nikon digital camera DS-Fi1 and Olympus SZX9 stereomicroscope were used for taking pictures of the dissections, which were then processed in Combine ZP and Corel PHOTO-PAINT X3 programs to enhance focus and improve quality. Genital dissections are kept in glycerol vials pinned under corresponding specimens. Genital terminology follows largely Razowski (1996) and Klots (1956). Wing venation follows the Comstock-Needham system. Head microstructures were examined under an Olympus SZX9 stereomicroscope. Adults were photographed with an Olympus E-500 digital camera and plates were composed with Adobe PhotoShop 8. The following abbreviations were used: FW, forewing; HW, hindwing; D, dorsum; V, venter. Types and comparison material was examined in major European and American museums and private collections, as listed below:

NHMUK: Natural History Museum, London, UK

EML: Collection of Eduardo Marabuto, Lisbon, Portugal

INBio: Instituto Nacional de Biodiversidad, San José, Costa Rica

CEP-MZUJ: Nature Education Centre (formerly Zoological Museum), Jagiellonian University, Kraków, Poland

PBF: Collection of Pierre Boyer, Le Puy Sainte Réparade, France (to be deposited in CEP-MZUJ)

INNY: Collection of Ichiro Nakamura in Williamsville, NY, USA

MNKB: Museum für Naturkunde, Berlin, Germany

NMNH: National Museum of Natural History, Smithsonian Institution, Washington, USA

SMTD: Seckenberg Museum für Tierkunde, Dresden, Germany

### Molecular analysis

For molecular analysis, two legs were removed from *Oxeoschistus cothon* (three specimens); *O. hilara* (Bates 1865) (two specimens); *O. tauropolis* (one specimen); *O. isolda* Thieme 1907 (one specimen); *O. euriphyle* (one specimens); and *Pronophila timanthes* Salvin 1871 (one specimen) (Supplementary Table 1). Samples were detached and preserved in 1 ml of ethanol, prior to mounting. DNA was extracted using Macherey-Nagel’s Nucleospin Tissue extraction kit, following the manufacturer’s protocol. Amplification of mitochondrial gene COI was performed in 20 μl volume, using LCO1490 and HCO2198 primers (Folmer *et al*^1994^), with the following PCR cycling protocol: 95°C for 5 min, 40 cycles of 94°C for 30 s, 50°C for 30 s, 72°C for 1 min 30 s and a final extension period of 72°C for 10 min. PCR products were sent for purification and sequencing to Macrogen (Amsterdam, Netherlands). Additional 25 sequences of six species were imported from GenBank and BOLD Systems databases: *Oxoeschistus cothon*; *O. tauropolis*; *O. leucospilos* Staudinger, 1876; *O. pronax* (Hewitson, [1850]); *O. puerta* (Westwood, 1851); and *O. euriphyle*. Final matrix involved 34 nucleotide sequences with a total of 616 positions in final dataset. All sequences were aligned manually in Bioedit, version 7.0.9.0. (Hall 1999). Evolutionary analyses were conducted in MEGA X (Kumar *et al*^2018^). Pairwise distances were calculated using the Maximum Composite Likelihood model (Tamura *et al*^2004^) and pairwise deletion option. A Maximum Likelihood tree was inferred using Tamura 3-parameter (Tamura 1992) model and partial deletion option. The branch support for internal nodes was measured using 1000 rapid bootstrap replicates. The final tree was edited in Corel DRAW 2018 to enhance picture quality. Analyses were performed in Molecular Laboratories of the Nature Education Centre and the Institute of Zoology and Biochemical Science of the Jagiellonian University. Sample data, along with GenBank accession numbers, are compiled in Supplementary Table 1.

## Results

### *Oxeoschistus tauropolis tauropolis* (Westwood, 1851)

*Pronophila tauropolis* Westwood, 1851: 358, pl. 66, Fig 1.

*Pronophila tauropolis* Westwood; Hewitson 1862: 14; Bates, 1864: 164; 1866: 157; Butler 1868: 180.

*Oxeoschistus tauropolis* (Westwood); Kirby 1871: 106; Godman and Salvin 1881: 108; Staudinger, 1888: 234; Lamas *et al*^2004^: 211.

*Dioriste tauropolis* (Westwood); Thieme, 1906: 171; Weymer 1911: 270, Fig 58c; Gaede 1931: 515; DeVries 1987: 278, pl. 49, Figs 2, 5.

*Pronophila laetific* Bates 1865: 164; Gaede 1931: 515 (as syn.).

Examined material: GUATEMALA: 31♂ and 10♀: Suchitepequez, Los Tarrales, Vesubio–Atitlán, 1000–1050 m, 14°31′16″N/91°11′34″W, 28.IX–21.X.2008, T. Pyrcz leg., CEP-MZUJ; 1♂ and 1♀: El Progreso, la Unión Barrios, 1500 m, 15°12′9″N/90°12′5″W, T. Pyrcz leg., CEP-MZUJ; 3♂ and 2♀: Suchitepequez, Los Tarrales, Volcán Atitlán versant sud, 900–1200 m, 14°31′16″N/91°11′34″W, 19.X.2005, P. Boyer leg., PBF.; 2♂ and 1♀: Suchitepequez, Los Tarrales, Volcán Atitlán versant sud, 1200–1500 m 18.X.2005, 14°32′36″N/91°10′35″W, P. Boyer leg., PBF; 1♀: El Progreso, la Unión Barrios, près de Purulha 1600 m, P. Boyer leg., PBF; 1♂: Suchitepequez, Los Tarrales, Vesubio–Atitlán, 1250 m, 28.X.2011, I. Nakamura leg., INNY; 1♂: Suchitepequez, Los Tarrales, Vesubio–Atitlán, 1400–1500 m, 14°32′36″N/91°10′35″W, 28.X.2011, I. Nakamura leg., INNY; 3♂: Suchitepequez, Los Tarrales, Vesubio–Atitlán, 800–1400 m, 14°32′36″N/91°10′35″W, 18–20.VI, 2015, I. Nakamura leg., INNY; 1♂ and 1♀: Huehuetenango, Chacula, 15°58′30″N/91°39′02″W, 1438 m, 8.X.2017, I. Nakamura leg., INNY. MEXICO: 3♂ and 1♀: Oaxaca, Sierra de Juarez, Metates, 1400 m, 2005, ex coll. M. Dottax, local collector leg., prep. genit. 02_23.08.2017/K.Florczyk; 2♀: Oaxaca, La Espranza, 1500 m, III.2003, local collector leg., CEP-MZUJ; 7♂ and 1♀: Veracruz, Santiago Tuxtla, 2007, ex coll. M. Dottax, local collector leg., prep. genit. 03_23.08.2017/K.Florczyk, CEP-MZUJ; Chiapas, Santa Rosa Comitan, IX.1969, ex coll. Henri Descimon, PBF; 1♂: Oaxaca, Metates, 700 m?, 2005, local collector leg., PB; Additional material: MEXICO: 1♂: Puebla, la Ceiba, VIII.1996, local collector leg., PBF; 1♂: Puebla, Patla, VI.1996, local collector leg., PB.; 1♂: no data, 28.VII.1985, local collector leg., PBF; 1♂ and 1♀: Puebla, Xicotepec de Juarez, VI–VIII.2005, ex coll. M. Dottax, local collector leg., CEP-MZUJ; 2♂: no data, ex coll. Staudinger & Bang-Haas, CEP-MZUJ.

#### Remarks

*O. tauropolis* was described from Oaxaca in southern Mexico (Type in NHMUK [examined], illustrated: www.butterfliesofamerica.com). *Pronophila laetifica* (Bates 1864), described from Polochic Valley (Caribbean slopes, Sierra de Cuchumatanes) in Guatemala (Type in NHMUK: examined; illustrated: www.butterfliesofamerica.com), was later recognized as its subjective junior synonym (Gaede 1931), an opinion upheld by all the subsequent authors. Although the type locality of *O. tauropolis* is vague, referring to a large Mexican state not directly bordering on Guatemala, we are inclined to concur with this decision since the two types are nearly identical externally. We need to point out, however, that the specimens from the Pacific slopes in Guatemala (Sierra Madre) are markedly smaller, with the inner edge of the HW yellow median patch straight from costa to outer section of discal cell, compared to, in most of the cases, the notched edge in discal cell in the Cuchumatanes or Oaxacan specimens. *O. tauropolis* is widely distributed throughout Mexico (Oaxaca, Chiapas, Veracruz, Puebla) and some populations possibly represent separate subspecies (Lamas *et al*^2004^). We refrain from taking any taxonomic action concerning Mexican populations here since we do not have sufficient material from Mexico for comparison. *O. tauropolis tauropolis* also occurs in Honduras where the local population found in the Parque Nacional Cusuco does not differ morphologically from the nominate subspecies. It is also found in Nicaragua (Matagalpa) (Maes 1997).

In Guatemala, on the Pacific slopes *O. tauropolis* occurs in clearings and near thickets of bambusaceous herbs in remnants of cloud forest and in semi-arid subtropical pine forests at 1000–1500 m. In Cobán, on the Caribbean slopes we found it at some 1400–1600 m in forest clearings and in secondary forests.

### *Oxeoschistus tauropolis mitsuko* Pyrcz & Nakamura, n. ssp. (Figs 1a–d, 5f, 6c)

Type locality: Costa Rica, San José, University for Peace Reserve, Rodeo

**Fig 1:**
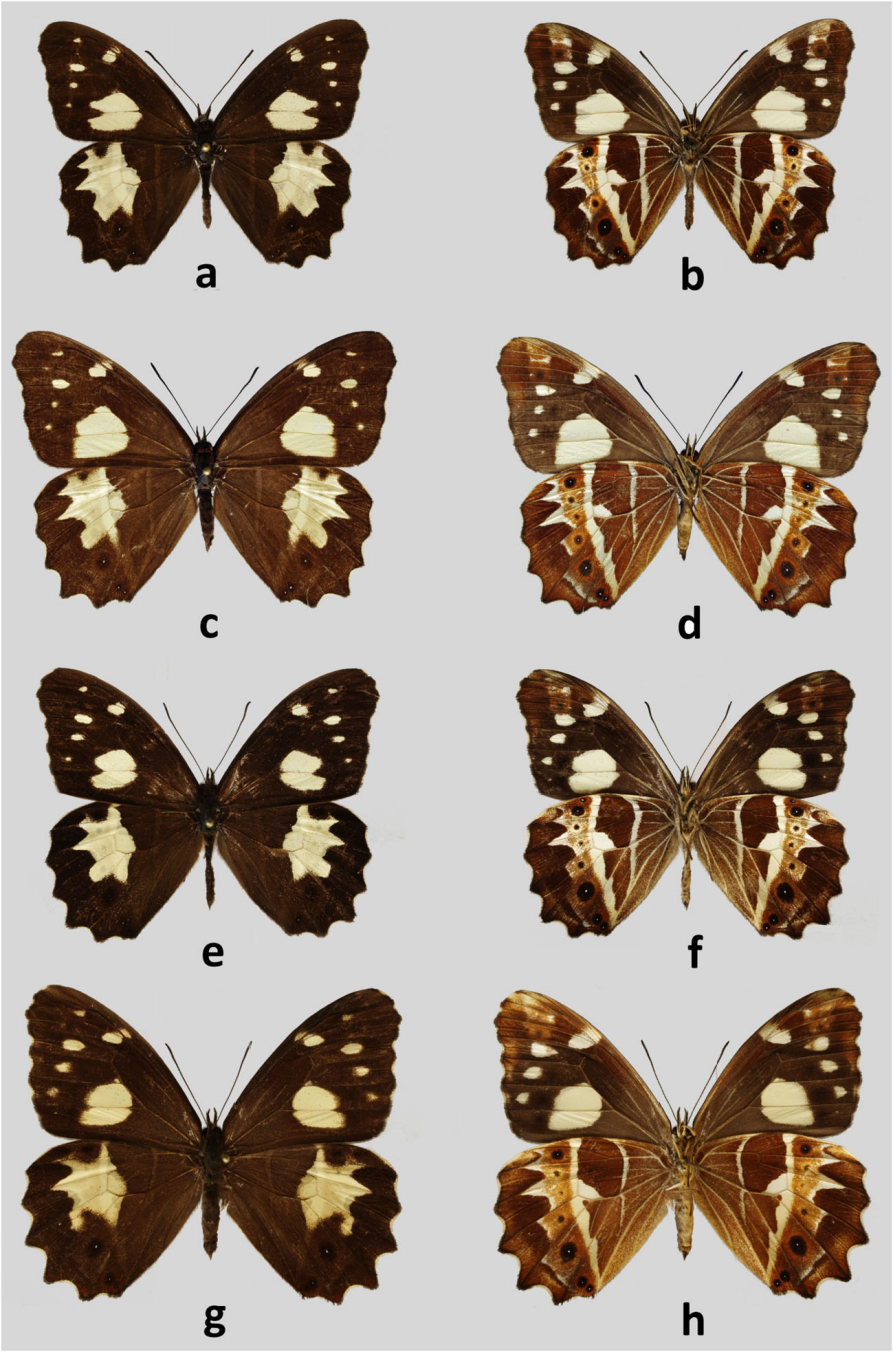
Adults. **a***Oxeoschistus tauropolis mitsuko*, male, Holotype, upperside, El Rodeo, Costa Rica. **b***Oxeoschistus tauropolis mitsuko*, male, Holotype, underside, El Rodeo, Costa Rica. **c***Oxeoschistus tauropolis mitsuko*, female, Paratype, upperside, San José, Costa Rica. **d***Oxeoschistus tauropolis mitsuko*, female, Paratype, underside, San José, Costa Rica. **e***Oxeoschistus tauropolis tauropolis*, male, upperside, Los Tarrales, Guatemala. **f***Oxeoschistus tauropolis tauropolis*, male, underside, Los Tarrales, Guatemala. **g***Oxeoschistus tauropolis tauropolis*, female, upperside, Los Tarrales, Guatemala. **h***Oxeoschistus tauropolis tauropolis*, female, underside, Los Tarrales, Guatemala

Type material: HOLOTYPE ♂: COSTA RICA, Prov. San José, Univ. for Peace Reserve, Rodeo, 1000 m, 9°54′42″N 84°16′54″W, 29.VIII.2012, I. & M. Nakamura leg., prep. genit. 04_23.08.2017/K.Florczyk, CEP-MZUJ; PARATYPES (13♂ and 3♀): COSTA RICA: 1♂: same data as the Holotype, CEP-MZUJ; 1♂: Prov. San José, Res. Ecol. L. Oviedo, UCR, San Pedro, 30.VIII.2004, I. & M. Nakamura leg. INNY; 1♂: Prov. Heredia, Finca Bernal, 1600 m, 4 km NE of San Rafael, 10°02′25″N 84°04′37″W, 23.II.2004, I. Nakamura & J. Corrales leg., INNY; 1♂: Prov. San José, Res. Ecol. L. Oviedo, UCR, San Pedro, 25.VIII.2004, I. & M. Nakamura leg., INNY; 1♂: Prov. San José, Res. Ecol. L. Oviedo, UCR, San Pedro, 20.IX.2004, I. Nakamura leg., INNY; 1♂ and 1♀: Prov. San José Res. Ecol. L. Oviedo, UCR San Pedro, 19.II.2004, I. Nakamura & K. Nishida leg., INNY; 1♂: Prov. Cartago, Cerros de la Carpintera, 13,501–500 m, 14.V.2005, I. & M. Nakamura & K. Nishida leg. INNY; 1♀: Prov. San José, Res. Ecol. L. Oviedo, UCR, San Pedro, 7.VI.2005, I. Nakamura leg., INNY; 1♀: Prov. San José, Centro Deportivo, UCR, San Pedro, 27.III.2006, I. Nakamura & K. Nishida leg., INNY; 1♂: Prov. San José, Res. Ecol. L. Oviedo, UCR, San Pedro 28.III.2006, I. & M. Nakamura leg. on banana trap, INNY; 1♂: Prov. San José, Centro Deportivo, UCR, San Pedro, 29.X.2006, I. Nakamura leg., CEP-MZUJ; 1♂: Prov. San José, Univ. for Peace Reserve, Rodeo, 900–1000 m, 9°54′42″N 84°16′54″W, 5.XI.2006, I. Nakamura leg., INNY; 1♂: Prov. San José, Univ. for Peace Reserve, Rodeo, 1000 m, 19.VIII.2008, I. Nakamura leg., CEP-MZUJ; 2♂: Prov. San José, Univ. for Peace Reserve, Rodeo, 1000 m, 21.IX.2011, I. &M. Nakamura leg., 1♂ INNY, 1♂ CEP-MZUJ. Some paratypes will be deposited in NMNH Smithsonian Institution, Washington D.C., and McGuire Center for Lepidoptera and Biodiversity, Florida Museum of Natural History.

#### Diagnosis

The new subspecies is not easily recognized externally from the nominate, except for the smaller size, even though quite variable within *O. tauropolis tauropolis*, and the slightly wider FWD median light yellow patch, which extends more distally than in any examined specimen of the nominate subspecies, in Cu2–2A reaching the origin of vein Cu2. Moreover, in most individuals of both sexes, the FWD yellow patch includes a tiny spot at the base of M3–Cu1. This occurs in females of the nominate subspecies, but rarely in males. The most consistent difference is, however, in the male genitalia, as described below.

#### Description

MALE (Fig 1a, b): head—eyes chocolate brown, hairy; labial palpi two times the length of head, milky white laterally, dark brown through the middle; antennae 2/5 the length of costa, shaft slender, club elongated, slightly wider than shaft, dark brown. Thorax: dorsally and ventrally dark brown, legs dull brown. Abdomen: dorsally dark brown, laterally and ventrally dull brown. Wings: FW length: 24–25 mm (*n* = 13); apex blunt, outer margin slightly produced in subapical area, between R4 and M2, from M2 to tornus slightly wavy. FWD blackish brown, a large pale yellow oval patch in median area in CuA1–CuA2, CuA2–1A+2A and the base of M3–Cu1, with a prominent notch along vein CuA2. A series of small patches of same colour is present, one oval in postdiscal area at the base of veins R+M1, one triangular in subapical area and three oval in M2–M3, M3–CuA1 and CuA1–CuA2, progressively smaller. HW oval with an undulated outer margin; HWD ground colour blackish brown, with a large median-submarginal pale yellow patch extending from costa to vein CuA2, entering discal cell, with a nearly straight inner margin and zigzagging outer margins, enclosing a rounded brown spot near costal margin, 3-min black dots with white pupils near tornus, one in CuA1-CuA2 and two in CuA2–1A+2A. FWV colour patterns similar to the upperside, but ground colour paler and lighter, and yellow patches slightly larger, with the subdued notch along vein CuA2, veins in basal area marked with yellow, and a chocolate brown overcast in apical area and an additional black pupil in subapical area. HWV ground colour chocolate brown with a pattern consisting of one postbasal, straight, narrow yellow band extending from costa to vein 1A+2A, and a wide postdiscal to submarginal band of the same colour with a roughly straight inner edge except for a prominent notch protruding into discal cell, and zigzagging outer edge reflecting the shape of the yellow patch from the upperside, with a series of rounded 6–7 ocelli of different sizes and colours, the largest of which, black with a white pupil and ringed with orange in CuA1–CuA2, the smaller, barely noticeable in M2–M3. Male genitalia (Fig 5f): differ from the nominate subspecies (Fig 5g–i) in the shape of the valvae, in particular the narrower apical part without the protrusion pointing upwards present invariably in all the specimens of the remaining populations. FEMALE (Fig 1c, d): sexual dimorphism little marked, with the female larger (FW length 27–30 mm), lighter on both the upper and underside, most notably on the HWV where instead of male’s chocolate brown, female’s ground colour is rufous brown, and on the FWD where the large yellow patch is larger with a less pronounced basal notch on the vein CuA2. Female genitalia (Fig 6c): similar to the nominate subspecies (Fig 6a, b) except for the larger, sclerotized basal plate of the posterior apophyse.

#### Etymology

This subspecies is dedicated to Mitsuko Nakamura, the wife of Ichiro Nakamura who joined him in many of his collecting trips.

#### Remarks

There has been some controversy about the occurrence of *Oxeoschistus tauropolis* in Costa Rica. Its reports from this country go back in time to Godman and Salvin’s “Biologia Centrali-Americana” (1881) who referred to some specimens collected by Rogers. The authors at the same time doubted whether Rogers’ specimens really came from Costa Rica or were perhaps mislabelled and actually came from Guatemala where Rogers also collected. Nonetheless, this doubtful report was perpetuated without any cross-checking by all subsequent authors for over a hundred years. Even the possibility that the two taxa, Costa Rican *O. cothon* and more northerly *O. tauropolis*, were, in fact, conspecific was considered (Gaede 1931). DeVries (1987) cited *O. tauropolis* from Costa Rica but was clearly hesitant about its status and relations with *O. cothon*. Again, some later authors cited *O. tauropolis* based on DeVries’s book without any cross-checking (Chacón & Montero 2007, Duong & Junger 2015). The presence of *O. tauropolis* in Costa Rica was eventually confirmed by Nishida *et al* (2009) who reported a population within the capital city of San José. Although DeVries (1987) included Panama as a part of its range, its occurrence in this country is doubtful. The Gordon Small collection at the Smithsonian NMNH does not contain any specimens from Panama (R. Robbins, pers. comm.) and two experts on Panamanian butterflies have not seen an individual in spite of years of collecting there (J. MacDonald & A. Thurman, pers. comm.).

*Oxeoschistus tauropolis mitsuko* is found so far at an altitude of approximately 1000 m which is the elevation it is usually reported at in other areas. Currently known localities are all in and around the Central Valley of Costa Rica (Fig 8). DeVries (1987) claims that *O. cothon* and *O. tauropolis* fly within approximately the same altitudinal band. In our experience, *O. tauropolis* occurs, however, at lower elevations than *O. cothon*. So their distribution in Costa Rica is rather parapatric along an elevation gradient than sympatric. Nishida *et al* (2009) list *Guadua angustifolia* as its potential host plant in San José. In Guatemala *O. tauropolis* is often associated with bamboos such as *Erytrostachys*, contrary to *O. hilara* which feeds exclusively on *Chusquea* (DeVries 1987, Maes 1997).

The population occurring in Costa Rica differs consistently from the specimens of *O. tauropolis* from Guatemala or Mexico by some trait of male genitalia, in particular the shape of valvae, consistently stable throughout the range of the nominotypical subspecies indicating it is a geographical race with fixed phenotypic characters. COI data are available now for only one specimen of the nominate subspecies which is premature to discuss genetic distances between the two subspecies. On the other hand, the taxon from Costa Rica does not present enough morphological differences, particularly at the genital level which would impede mating, nor in wing colour patterns which would prevent species recognition, so it does not merit a separate status.

The most immediate way to separate *O. tauropolis* from *O. cothon* is by comparing their hindwing ventral patterns. The yellow patch situated at distal end of discal cell in *O. tauropolis* is narrow, roughly triangular, and restricted to the lower part of the cell along cross-vein M3–Cu1A, whereas in *O. cothon* it is large, roughly rectangular and aligned with the basal edge of the upper part of postdiscal yellow band. There are other distinctive characters as well, in particular the much more indented distal edge of the yellow postdiscal band, especially along veins M1 and M2. In *O. cothon*, the large median yellow patch is usually considerably larger and more compact than in *O. tauropolis*. Besides, *O. cothon* is the larger of the two, which is evident by comparing their FW lengths, an average 3 mm longer in *O. cothon*.

### *Oxeoschistus cothon* Salvin, 1869

#### *Oxeoschistus cothon* f. *cothonides* Grose-Smith, 1869, n. stat (Figs 2a–h, 5d, 6e, f)

**Fig 2:**
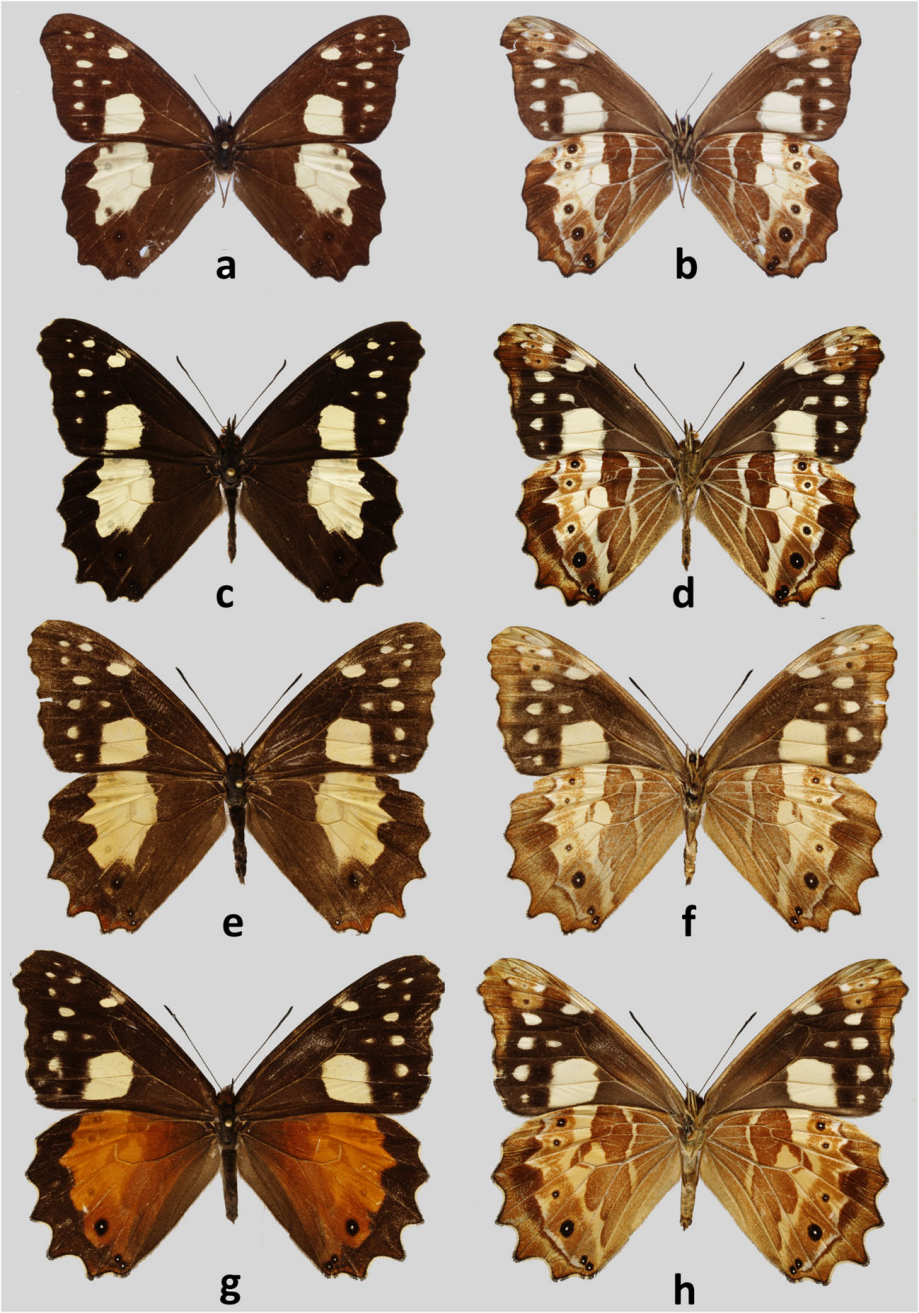
Adults. **a***Oxeoschistus cothon*, male, upperside, Chirriquí, Costa Rica. **b***Oxeoschistus cothon*, male, underside, Chirriquí, Costa Rica. **c***Oxeoschistus cothon*, male, upperside, Cerro de la Muerte, Costa Rica. **d***Oxeoschistus cothon*, male, underside, Cerro de la Muerte, Costa Rica. **e***Oxeoschistus cothon*, female, upperside, Cerro de la Muerte, Costa Rica. **f***Oxeoschistus cothon*, female, underside, Cerro de la Muerte, Costa Rica. **g***Oxeoschistus cothon f. cothonides*, female, upperside, Cerro de la Muerte, Costa Rica. **h***Oxeoschistus cothon, f. cothonides*, female, underside, Cerro de la Muerte, Costa Rica

*Oxeoschistus cothon* Salvin, 1896: 413.

*Oxeoschistus cothon* Salvin; Godman & Druce, 1874: 338; Godman and Salvin 1881: 108, Figs 10, 11; Staudinger, 1888: 234, Fig 84; Lamas *et al*^2004^: 211.

*Dioriste cothon* (Salvin); Thieme, 1906: 172; Weymer 1911: 270; Gaede 1931: 516; DeVries 1987: 279, pl. 49, Figs 3, 6.

*Oxeoschistus cothonides* Grose-Smith 1896: 241.

*Oxeoschistus cothonides* Grose-Smith 1896: 1900: Fig 3; Lamas *et al*^2004^: 211.

*Dioriste cothonides* (Grose-Smith); Thieme, 1906: 173; Weymer 1911: 270, Fig 58d; Gaede 1931: 515; DeVries 1987: 279, pl. 49, Fig 4; d’Abrera, 1988: 811.

Material examined: COSTA RICA: 2♂: Prov. San José, División–Santa Eduviges, 1700–2000 m, 9°29′37″N/83°43′97″W, 29.II.2016, T. Pyrcz leg., CEP-MZUJ; 1♂: Prov. San José, División–Santa Eduviges, 1700–1850 m, 9°29′37″N/83°43′97″W, 08.III.2016, T. Pyrcz leg., CEP-MZUJ; 1♂: Prov. San José, División–Santa Eduviges, 1900–2050 m, 9°29′37″N/83°43′97″W, 10.III.2016, T. Pyrcz leg., prep. genit. 365_11.04.2016/J.Lorenc, CEP-MZUJ; 2♂: Prov. San José, División–Santa Eduviges, 1900–2050 m, 9°29′37″N/83°43′97″W, 11.III.2016, T. Pyrcz leg., prep. genit. 366_11.04.2016/J.Lorenc, CEP-MZUJ; 1♂: Prov. San José, División–Santa Eduviges, 1900–2050 m, 9°29′37″N/83°43′97″W, 12.III.2016, T. Pyrcz leg., CEP-MZUJ; 2♂: Prov. San José, División–Santa Eduviges, 1900–2050 m, 9°29′37″N/83°43′97″W, 13.III.2016, T. Pyrcz leg., CEP-MZUJ; 1♀: Prov. San José, División–Santa Eduviges, 1900–2050 m, 9°29′37″N/83°43′97″W, 11.III.2016, T. Pyrcz leg., CEP-MZUJ (f. *cothon*); 2♀: Prov. San José, División–Santa Eduviges, 1900–2050 m, 9°29′37″N/83°43′97″W, 11.III.2016, T. Pyrcz leg., CEP-MZUJ (f. *cothon*); 2♀: Prov. San José, División–Santa Eduviges, 1900–2050 m, 9°29′37″N/83°43′97″W, 13.III.2016, T. Pyrcz leg., CEP-MZUJ (f. *cothon*); 1♀: Prov. San José, División–Santa Eduviges, 1800–2050 m, 9°29′37″N/83°43′97″W, 10.III.2016, T. Pyrcz leg., CEP-MZUJ (f. *cothon*); 3♀: Prov. San José, División–Santa Eduviges, 1900–2050 m, 9°29′37″N/83°43′97″W, 11.III.2016, T. Pyrcz leg., CEP-MZUJ (f. *cothonides*); 2♂: Prov. Cartago, Parque Nat. Tapanti, ca.1400 m, 9°44′0″N/83°46′46″W, 3.VI.2005, I. & M. Nakamura, K. Nishida & A. Damaceno leg., INNY; 6♂: Prov. San José, Bajo La Hondura, 1150–1450 m, 10°03′37″N/83°58′55″W, 13.VI.2005, I. Nakamura leg., INNY; 1♂: Prov. San José, 4 km SW of División, 1800–2100 m, 9°29′31″N/83°44′21″W, 26.IV.2006, I. & M. Nakamura & M. Posla leg., INNY; 1♂: Prov. Heredia, End of Calle Zurqui, 1680 m, 10°03′01″N/84°01′24″W, 24.X.2006, I. Nakamura leg., INNY; 1♂: Prov. San José, 4 km SW of División, 1800–2100 m, 9°29′31″N/83°44′21″W, 27.X.2006, I. Nakamura leg., INNY; 1♂: Prov. Cartago, Above Muńeco, Orosi Valley, 1350–1500 m, 9°46′23″N/83°54′14″W, 2.XI.2006, M. Posla & Doninelli leg., INNY; 3♂: Prov. Cartago, Alto Belén, above Muńeco, Orosi Valley, 1500–1700 m, 9°45′56″N/83°54′04″W, 2.XI.2006, I. Nakamura leg., INNY; 1♂: Alto Belén, same as sbove, 9.XI.2006, I. Nakamura leg., INNY; 3♂: Alto Belén, same as above, 14.XI.2006, I. Nakamura leg., INNY; 3♂: Prov. Heredia, End of Calle Zurqui, 1680 m, 10°03′01″N 84°01′24″W, 24.VIII.2007, I. & M. Nakamura leg., INNY; 1♂: Prov. Heredia, End of Calle Zurqui, as above, 1.IX.2007, I. Nakamura leg., INNY; 1♂: Prov. Heredia, End of Calle Zurqui as above, 6.IX, 2007, I. & M. Nakamura leg., INNY; 1♀: Prov. San José, Bajo La Hondura, 1150–1450 m, 10°03′37″N 83°58′55″W, 19.IX.2007, I. Nakamura leg., INNY (f. *cothonides*); 2♂ and 1♀: Prov. Cartago, Alto Belén, above Muńeco, Orosi Valley, 1500–1700 m, 26.IX.2007, I. Nakamura leg., INNY (f. *cothonides*); 1♂: Prov. San José, Bajo La Hondura, 10°03′37″N 83°58′55″W, 1150–1450 m, 31.VIII.2008, I. Nakamura leg., INNY; 3♂: Prov. Cartago, Alto Belén, above Muńeco, Orosi Valley, 1500–1900 m, 2.IX.2008, I. Nakamura leg., INNY; 2♂: Prov. San José, San Gerardo de Rivas to Llano Bonito on trail to Cerro Chirripo, 1870–2500 m, 19.II.2009, I. & M. Nakamura leg., INNY; 2♂: Prov. San José, Llano Bonito vicinity, on trail to Cerro Chirripo, 2500 m, 24.II.2009, I. & M. Nakamura & K. Nishida leg., INNY; 1♂: Prov. Cartago, Alto Belén, above Muńeco, Orosi Valley, 1500–1700 m, 21.VII.2010, I. Nakamura leg., INNY; 1♂: Prov. Cartago, Alto Belén, above Muńeco, Orosi Valley, 1500–1700 m, 30.VIII.2012, I. Nakamura leg., INNY; 1♀: Prov. Heredia, End of Calle Zurqui, 1680 m, 31.VIII.2012, I. Nakamura leg., INNY (f. *cothonides*); 3♂: Prov. Heredia, End of Calle Zurqui, 1680 m, 2, 3, 11.VI.2013, I. & M. Nakamura leg., INNY. PANAMA: 1♀: Chiriqui, ex Staudinger & Bang-Haas, CEP-MZUJ (f. *cothonides*).

##### Remarks

Validity of *Oxeoschistus cothonides* (Grose-Smith 1896) as a separate species was never questioned ever since its description over a hundred years ago. DeVries (1987), considered to be the primary and frequently the only source of information on Costa Rican butterfly fauna, taxonomy and ecology, referred to *O. cothonides* as a valid species occurring sympatrically with *O. cothon*. He even pointed out some putative ecological differences between the two. Some suspicion about its systematic status arose prior to this study because all the specimens of “*cothonides*” examined in extant historical scientific collections turned out to be females, including the holotype of Grose-Smith in London, then further historical specimens in Berlin and Dresden.

During our study, we confirmed that *cothonides* represents merely an individual form of the female of *Oxeoschistus cothon*. The two taxa, *cothon* and *cothonides* differ externally in a striking way by the colour of hindwing dorsum, covered almost entirely by a russet patch in *cothonides*, whereas in *cothon* it is marked by a large lemon yellow patch on the dark brown ground colour. Hindwing ventral surface, which is taxonomically very informative, is however identical in *cothon* and *cothonides*. Genital dissections of two females of *O. cothon* and *O. cothonides* (Fig 6e, f), and *O. euriphyle* and *O. tauropolis* for comparison (Fig 6g and a, respectively), were made showing no morphological differences between the former two, and significant differences, for example size and shape of the 8th tergite, compared to the latter. A preliminary molecular analysis (COI-based pairwise distance calculation) confirmed the above findings, showing that the genetic distances between *O. cothon* and *O. cothonides* are very low, ranging from 0 to 0.1 (Supplementary Table 2). Therefore, *Oxeoschistus cothonides* is formally relegated here to the status of a form of the female of *Oxeoschistus cothon*. Male genitalia of *O. cothon* (Fig 5d) are characterized by elongated valves with a serrate ampulla reminiscent of *O. hilara* (Fig 5b–c) but quite different from the massive, short valves of *O. tauropolis* (Fig 5f–i) terminated by a wide, blunt apex.

*Oxeoschistus cothon* was found to be relatively common in the lower part of the transects sampled in the Cordillera de Talamanca, in particular at some 1700–2000 m where the two forms of females occur syntopically. The altitudinal band provided for *O. cothon* by DeVries (1987), 1400–2000 m, is not entirely accurate. This species can be observed as high as 2500 m and perhaps as low as 1200–1300 m. Males are very active and patrol above clumps of bamboo. They occasionally go down to ground level to feed on decomposing organic matter, and if they do, it is during particularly humid and sunny days. Their flight is faster compared to other congeners.

### *Oxeoschistus hilara hilara* (Bates, 1865) (Figs 3c–h, 5h, 6h)

Type locality: Guatemala Pacific Slopes

**Fig 3:**
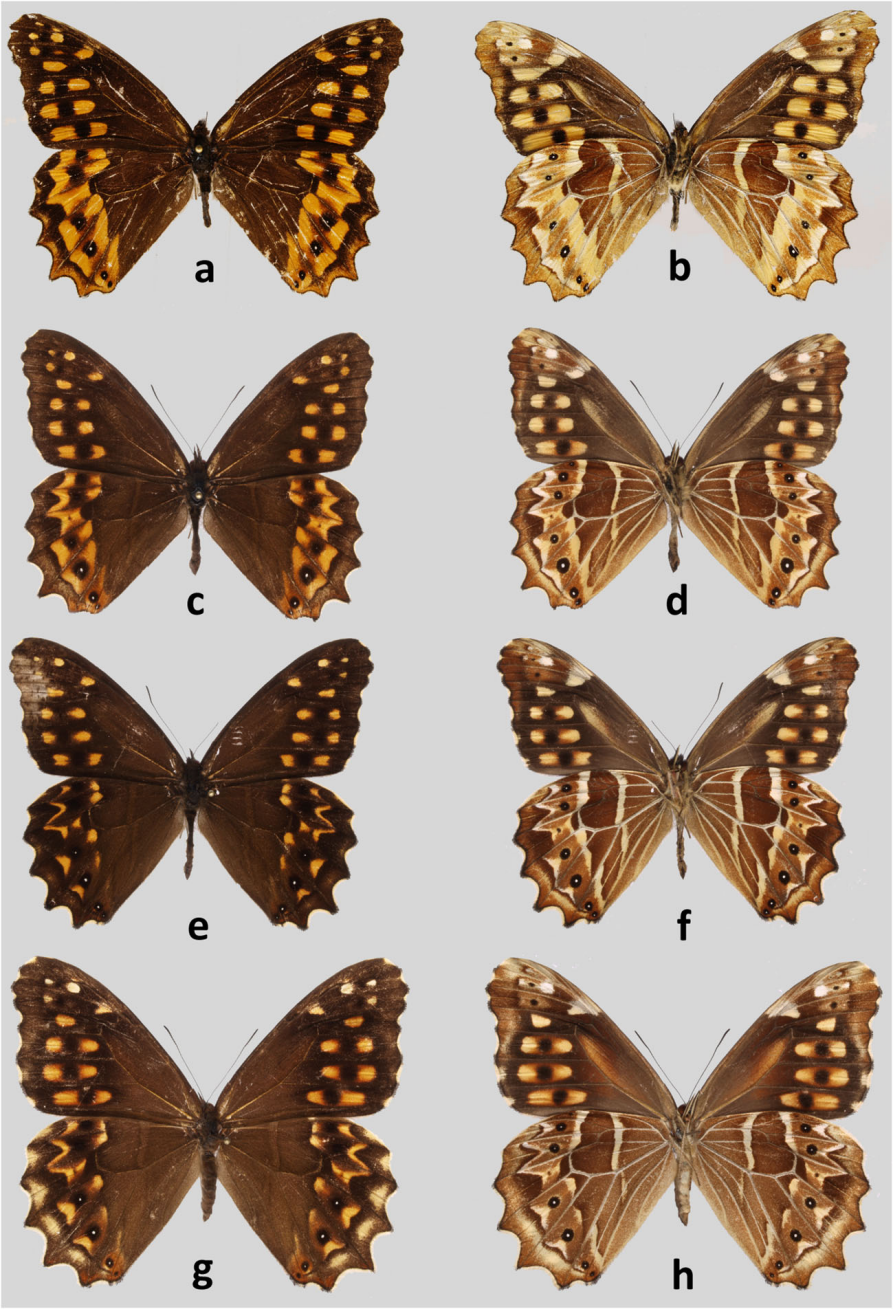
Adults. **a***Oxeoschistus hilara lempira*, male, Holotype, upperside, Honduras. **b***Oxeoschistus hilara lempira*, male, Holotype, underside, Honduras. **c***Oxeoschistus hilara hilara*, male, upperside, Chilascó, Guatemala. **d***Oxeoschistus hilara hilara*, male, underside, Chilascó, Guatemala. **e***Oxeoschistus hilara hilara*, male, upperside, Zuníl, Guatemala. **f***Oxeoschistus hilara hilara*, male, underside, Zuníl, Guatemala. **g***Oxeoschistus hilara hilara*, female, upperside, Zuníl, Guatemala. **h***Oxeoschistus hilara hilara*, female, underside, Zuníl, Guatemala

*Pronophila hilara* Bates, 1865: 178.

*Oxeoschistus hilara* (Bates); Butler, 1867: 268; Gaede, 1931: 516–517; Maes, 1997:5–6; Lamas, Viloria & Pyrcz, 2004: 211.

*Oxeoschistus hilarus* [sic] Bates; Butler, 1868: 180; Kirby, 1871: 106; Godman & Salvin, 1881: 107, pl. X, Figs 14, 15; Thieme, 1906: 186; Weymer, 1911: 272; d’Abrera, 1988: 810, Figs (male, dorsal/ventral); Pyrcz, 2004: 521 (all misspelling).

[*Oxeoschistus hilara* n. ssp. Llorente; Lamas *et al* 2004: 211.]

Examined material: HONDURAS: 2♂: Cerro Cantiles, Parque Nacional Cusuco, Sierra de Omoa, Merendón, Cortés, 15°31′00″N/88°14′00″W, 2000 m, 29.VII.2006, prep.genit.02_08.01.2007/T.Pyrcz, Marabuto leg., EML; GUATEMALA: 12♂ and 1♀: Prov. Quetzaltenango, Zunil, Las Georginas, 14°45′01″N/91°28′50″W, 2400–2450 m, 14–18.X.2005, T. Pyrcz leg., TWP, prep. genit. 05_09.11.2006/T.Pyrcz; 13♂: Prov. Suchitepequez, Reserva Los Tarrales, Vesubio via Volcán Atitlán, 01–21.X.2005, 1800–1950 m, 14°33′21″N/91°11′39″W, T. Pyrcz leg., CEP-MZUJ; 2♂: Prov. Saquatepequez, Volcán Acatenango, Cerro Sanay, 14°32′34″N/90°52′36″W, 2500–2550 m, 20.X.2005, T. Pyrcz leg., TWP; 1♂: Prov. Alta Verapaz, Cobán, Chilascó, 1800 m, 15°6′55″N/90°6′47″W, 11.X.2005, T. Pyrcz leg., TWP; 1♂: Suchitepequez, Los Tarrales, Volcán Atitlán versant sud, 1650–2000 m, 14°33′21″N/91°11′39″W, 1.X.2005, T. Pyrcz leg., PBF; 9♂: Quezaltenango, Las Georginas, 6 à 8 km sud est. de Zunil, 2300–2400 m, 14°45′5″N/91°28′52″W, 14–16.X.2005, P. Boyer leg., PBF; 1♂: Sololá, Corazón del Bosque, Novillero, Km 145 Interamericana, 2400–2500 m, 14°47′25″N/91°15′49″W, 19.VI.2016, I. Nakamura leg., INNY; 1♀: Quezaltenango, Fuentes Georginas, SE of Zunil, 2300–2450 m, 14^o^45′01″N/91^o^28′50″W, 22.VI.2016, J.A.Lopez Coyoy leg., INNY; 32♂ and 2♀: Quezaltenango, Ridge above Fuentes Georginas, SE of Zunil, 2735 m, 14°44′50″N/91°29′06″W, 21–25.VI.2016, I. Nakamura & J. A. Lopez Coyoy leg., INNY.

#### Remarks

Although the type locality of *Oxeoschistus hilara* (misspelled in most sources as *hilarus*) was defined by Bates vaguely as Guatemala, Godman & Salvin (1881) state that this species was described based on specimens they collected on the Volcán del Fuego (Antigua) and on the road from Quetzaltenango (Zuníl area) to the Pacific coast. *O. hilara* is found throughout southern Guatemalan Sierra Madre range on the slopes of the volcanoes Acatenango, Atitlán, Tolimán, Del Fuego, Del Agua and Santa María (Fig 9). It also occurs in the highlands of Cobán in north-central Guatemala. Maes (1997) discovered a population of the nominate *O. hilara* in Nicaragua, in the Matagalpa province in the western part of the country. Although the presence of *O. hilara* in Salvador has not been confirmed so far, currently known distribution pattern suggests its occurrence there, particularly along the border to Guatemala. *O. hilara* also occurs in Mexico, in the states of Chiapas, Oaxaca and Guerrero (de La Maza and de la Maza 1993). Weymer (1911) additionally lists it from Xantipa and Omilteme. Some Mexican populations apparently represent a separate subspecies of *O. hilara* (Lamas *et al*^2004^). We could not examine any specimens belonging to this race, therefore cannot comment on its status.

*Oxeoschistus hilara* presents considerable individual variation, in both males (Fig 3c–f) and females (Fig 3g, h), which affects mostly the expression of the yellow-orange pattern of the HWD. Although the specimens from the Sierra Madre in Guatemala have less orange markings on the HWD than the few from the Cobán area we could examine, as already pointed out by Godman & Salvin (1881), more sampling is needed to uncover a possible geographic pattern. The pattern of individuals collected in Nicaragua falls within the variation shown by the Guatemalan populations. *O. hilara* occurs in mid to high elevation cloud forests, from 1600 to 2600 m. It is locally common. It flies in the forest understory and is territorial in sunny gaps. Its colour pattern, unusual for the Neotropical Satyrinae, and also the behaviour is strongly reminiscent of the famous European *Pararge aegeria* (Linnaeus, 1758) (Davies, 1978). *O. hilara* is apparently an object of heavy predation from cloud forest birds. More than 50% of all examined individuals from Volcán Atitlán bear clear beak marks and serious wing damage, most often on the HW. Host plants of *O. hilara* are, as for most species of pronophilines, *Chusquea* bamboo (Maes 1997). Male genitalia (Fig 5b): Uncus 1/3 longer than tegumen, gently curved downwards; subunci 2/3 the length of uncus, wide in basal part, thin in apical part, slightly hooked upwards; saccus intermediate deep, flattened; valvae elongate, gradually narrowing from base to apex, dentate dorsally along apical half, no dorsal process, apex covered with short teeth like porcesses; aedeagus slightly shorter than tegumen + uncus, gently curved in median part. Female genitalia (Fig 6h) are most similar to *O. euriphyle* (Fig 6g) by the shape of corpus and ductus bursae, with somewhat shorter signa; however, the latter may be subject to some extent to individual variation.

### *Oxeoschistus hilara lempira* Pyrcz, n. ssp. (Figs 3a, b, 5c)

Type locality: Honduras, Prov. Lempira, Cerro Celaque.

Type material: Honduras, Holotype ♂: Honduras, Prov. Lempira, Cerro Celaque, 2550–2750 m, 14°31′35″N/88°40′29″W, 29.II.2008, P. Naks leg., prep. genit. 05_07.10.2009/J. Lorenc, CEP-MZUJ.

#### Diagnosis

This subspecies can be immediately recognised from the nominotypical *O. hilara* by the wide HWD rich yellow band; this pattern is also present in *O. euriphyle* whose band however extends onto FWD, whereas in *O. hilara hilara* it is broken on the FWD into a series of elongated patches.

#### Description

Male (Fig 3a, b): head—eyes chocolate brown, hairy; labial palpi two times the length of head, milky white laterally, dark brown through the middle; antennae 2/5 the length of costa, shaft slender, club elongated, slightly wider than shaft, dark brown. Thorax: dorsally and ventrally dark brown, legs dull brown. Abdomen: dorsally dark brown, laterally and ventrally dull brown. Wings: FW length—30 mm; apex blunt, outer margin slightly produced between R4 and M2, from M2 to tornus slightly wavy. FWD chestnut brown, five subapical oval apricot yellow patches, one postdiscal, two in R5–M1, one in M1–M2, one in M2–M3 slightly displaced basally compared to the latter, three larger, elongated patches of same colour, each with a dark rounded brown patch cutting them through the middle, in M3–Cu1, Cu1–Cu2 and Cu2–1A. HWD outer margin scalloped; hairy in basal one third and along anal margin, chestnut brown, a 3–4-mm-wide apricot yellow band, extending from postmedian to submarginal area, narrowing towards tornus, inner edge sharp but irregular, with a wide distal incision in M3–Cu1, and narrow incisions along veins Cu2 and 1A, outer margin irregular between M1 and Cu1, smooth between Cu1 and 1A, and a series of dark brown patches, two rectangular in M1–M2 and M2–M3 merging along vein M2, the remainder white pupiled, one rectangular in M3–Cu1 white pupiled, one oval in Cu1–Cu2 and two rounded in Cu2–1A considerably smaller than others, some pale yellow marginal scaling making up a diffused band from M3 to tornus. FWV colour pattern similar to the upperside, brown ground colour a shade lighter and duller, subapical patches larger and with diffused edges, milky white, two blackish dots in R5–M1 and M1–M2, patches in M3–Cu1, Cu1–Cu2 and Cu2–1A blonde yellow. HWV colour pattern similar to the upperside except for a pale yellow, 1-mm-wide median band extending from costa to vein 1A, the band extending from postmedian to submarginal area sandy yellow towards margins, pale orange in the middle, four submarginal patches smaller than on the upperside, blackish instead of dark brown, and all white pupiled, marginal area widely suffused with sandy yellow over the entire length. Male genitalia (Fig 5c): uncus nearly two times the length of tegumen shoulder, slightly arched downwards; subunci stout, 2/3 the length of uncus; saccus short, flattened; valvae elongate, narrow, gradually narrowing towards apex, finely dentate along dorsa; aedeagus approximately the length of valvae, straight. The genitalia of *O. hilara lempira* differ from the nominotypical subspecies in the longer uncus and proportionally smaller tegumen, and considerably narrower valvae. Female: unknown.

#### Etymology

This subspecies is named after the Central American Amerindian war captain Lempira of the Lencas tribe during the 1530s. A notable personality in Honduran history, he fought against the Spanish rule over the country. In Lenca language, the name means “The Lord of the Mountains”.

#### Remarks

*Oxeoschistus hilara lempira* n. ssp. was discovered in the isolated Cerro Las Minas massif within the Celaque National Park in central Honduras. Cerro Las Minas at 2814 m is the highest point of Honduras. Previously, a subspecies—*Pedaliodes napaea naksi* Pyrcz & Viloria—was described from the same area which was the first indication that the fauna of this area harbours endemic butterflies (Pyrcz & Viloria 2012). More focused sampling will possibly reveal further endemic elements of this region. It is impossible to ascertain currently about the distribution of this subspecies of *O. hilara*. Honduras topography is remarkable for the presence of several isolated but geographically restricted mountainous areas, archipelago-like, which promotes genetic divergence and the evolution of local populations with exclusive traits. Another population of *O. hilara* was discovered by F. Marabuto (pers. comm.) in the more northerly Merendón massif near the locality of San Pedro Sula. Based on the photographs, they are similar in many respects to the new subspecies, in particular in presenting a wide, uninterrupted yellowish hindwing band. We, however, abstain from associating it with *O. hilara lempira* n. ssp. until more exhaustive studies on its taxonomic status are performed.

### *Oxeoschistus euriphyle* Butler, 1872, stat. reinst. (Figs 4a–d, 5a, 6g)

**Fig 4:**
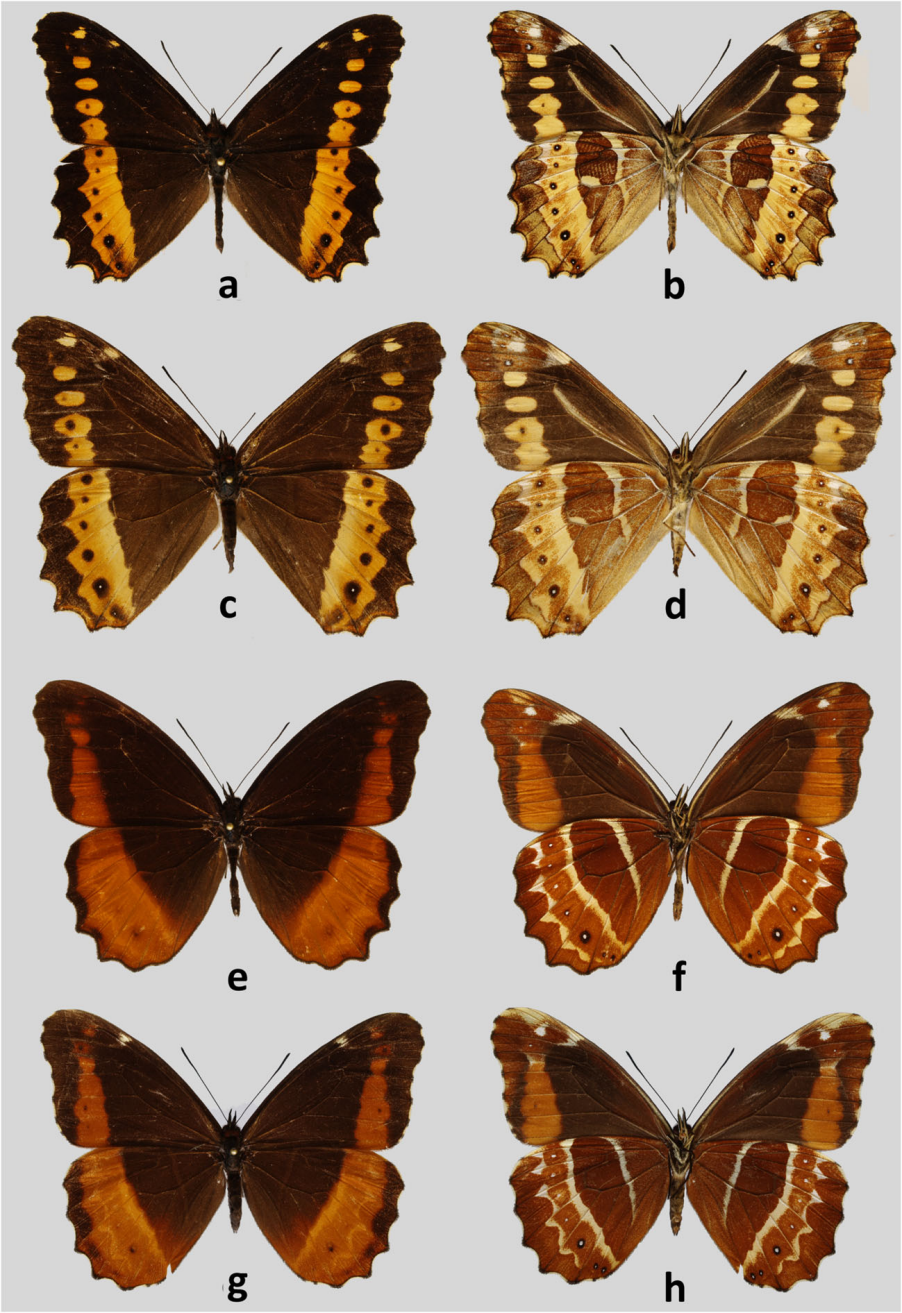
Adults. **a***Oxeoschistus euriphyle*, male, upperside, Cerro de La Muerte, Costa Rica. **b***Oxeoschistus euriphyle*, male, underside, Cerro de la Muerte, Costa Rica. **c***Oxeoschistus euriphyle*, female, upperside, Cerro de la Muerte, Costa Rica. **d***Oxeoschistus euriphyle*, female, underside, Cerro de la Muerte, Costa Rica. **e***Oxeoschistus puerta submaculatus*, male, upperside, Bajo La Hondura, Costa Rica. **f***Oxeoschistus puerta submaculatus*, male, underside, Bajo La Hondura, Costa Rica. **g***Oxeoschistus puerta submaculatus*, female, upperside, Alto Belén, Costa Rica. **h***Oxeoschistus puerta submaculatus*, female, underside, Alto Belén, Costa Rica

**Fig 5:**
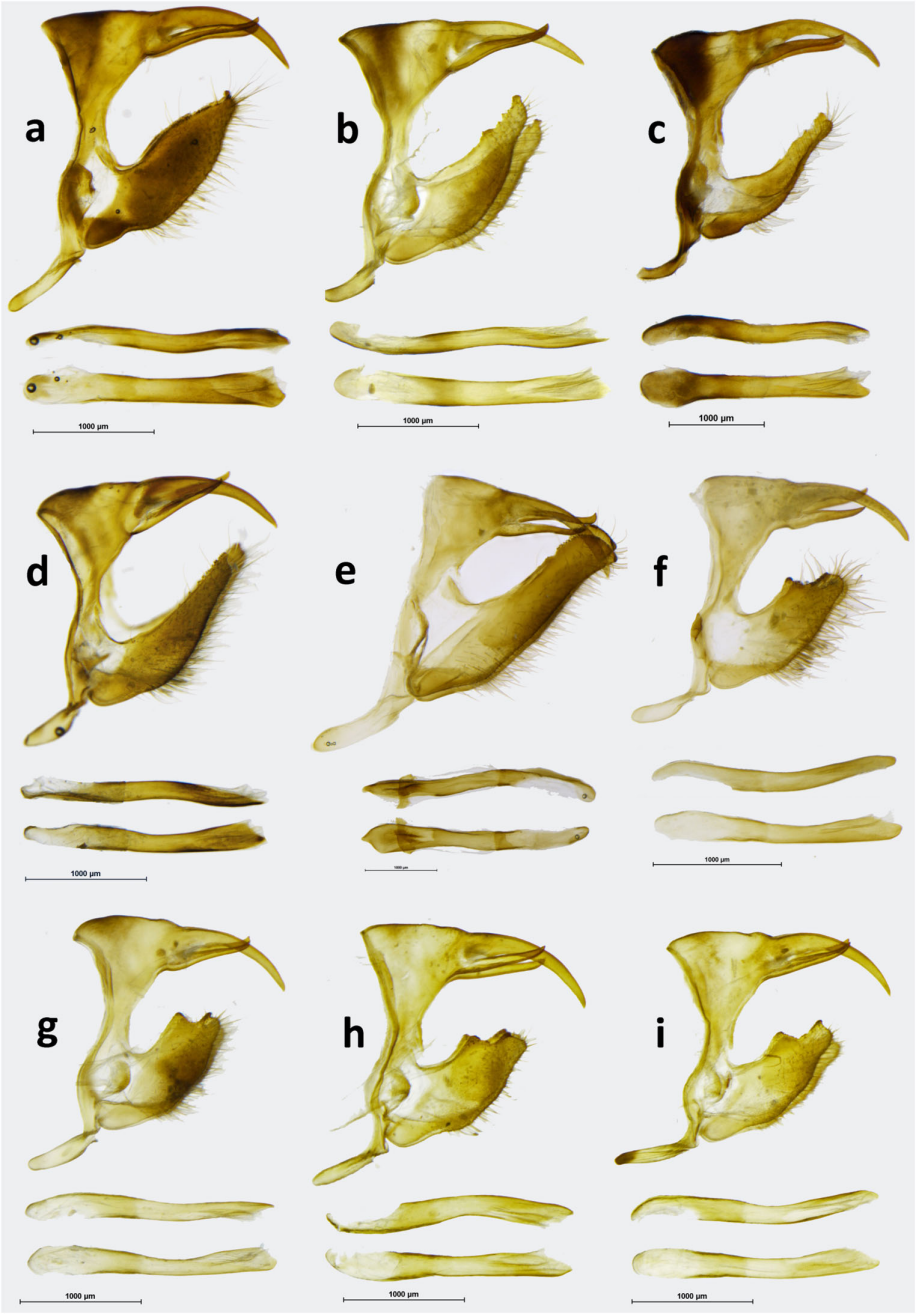
Male genitalia (in lateral view, aedeagus extracted, in lateral and dorsal view). **a***Oxeoschistus euriphyle*, Costa Rica, Cerro de la Muerte. **b***Oxeoschistus hilara hilara*, Guatemala, Zuníl. **c***Oxeoschistus hilara lempira*, Honduras, Cerro Celaque. **d***Oxeoschistus cothon*, Cerro de la Muerte, Costa Rica. **e***Oxeoschistus puerta submaculatus*, Bajo La Hondura, Costa Rica. **f***Oxeoschistus tauropolis mitsuko*, San José, Costa Rica. **g***Oxeoschistus tauropolis tauropolis*, Los Tarrales, Guatemala. **h***Oxeoschistus tauropolis tauropolis*, Tuxtla, Mexico. **i***Oxeoschistus tauropolis tauropolis*, Metates, Mexico

**Fig 6:**
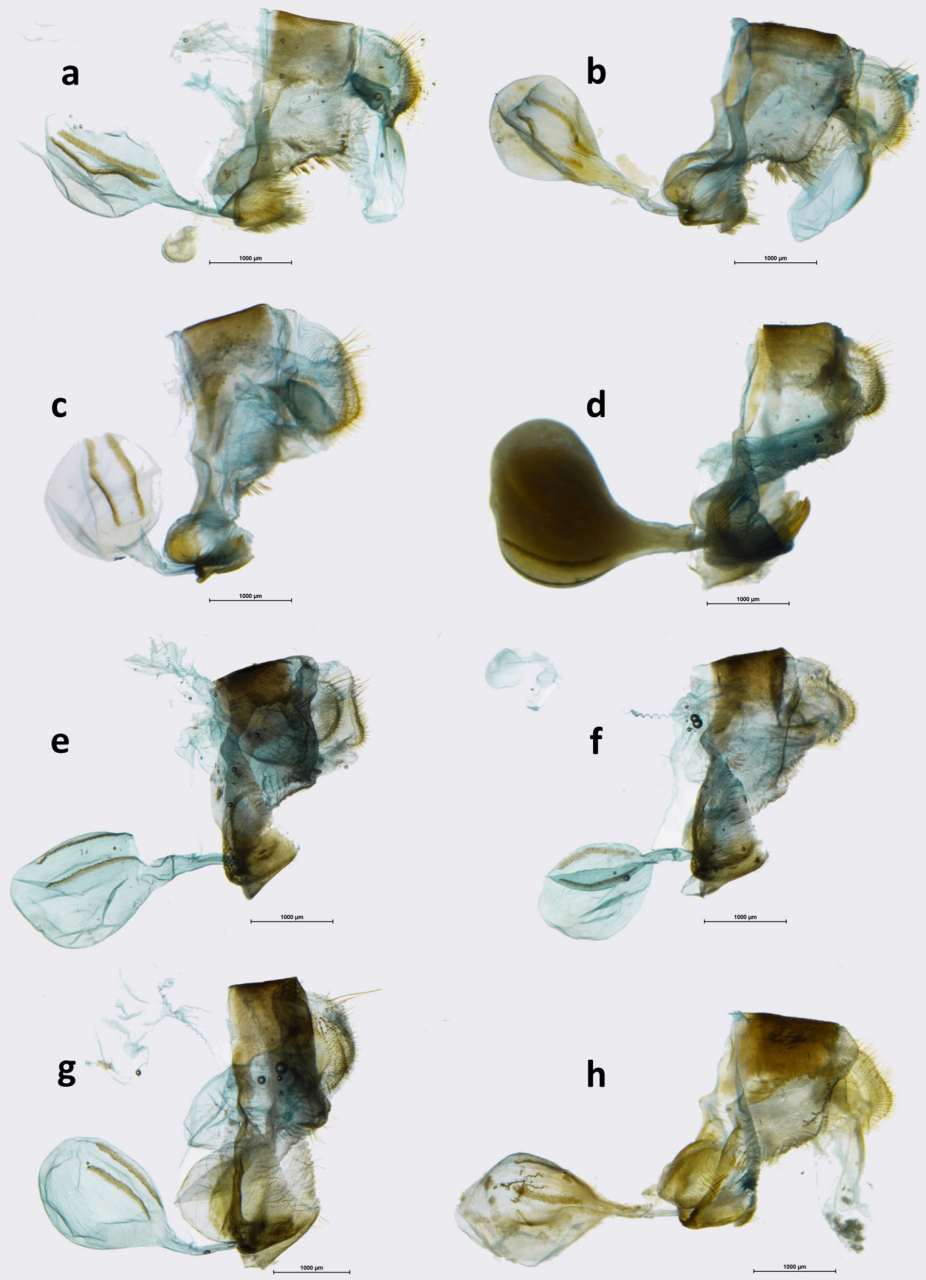
Female genitalia (in lateral view). **a***Oxeoschistus tauropolis tauropolis*, Los Tarrales, Guatemala. **b***Oxeoschistus tauropolis tauropolis*, Oaxaca, Mexico. **c***Oxeoschistus tauropolis mitsuko*, San José, Costa Rica. **d***Oxeoschistus puerta submaculatus*, Alto Belén, Costa Rica. **e***Oxeoschistus cothon*, Cerro de La Muerte, Costa Rica. **f***Oxeoschistus cothon* f. *cothonides*, Cerro de La Muerte, Costa Rica. **g***Oxeoschistus euriphyle*, Cerro de La Muerte, Costa Rica. **h***Oxeoschistus hilara hilara*, Zuníl, Guatemala.

*Oxeoschistus euriphyle* Butler, 1872: 73.

*Oxeoschistus euriphyle* Butler; Butler, 1874: 181; Butler & Druce, 1874: 338; Kirby, 1877: 710; Godman & Salvin, 1881: 107; DeVries, 1987: 279, pl. 50, Figs 2 (male, dorsal), 3 (female, ventral).

*Oxeoschistus euryphile* [sic] Butler; Godman & Salvin, 1901: 662; Thieme, 1906: 185–186; Weymer, 1911: 272, pl. 59, row a (all misspelled).

*Oxeoschistus eryphile* [sic] Butler; d’Abrera, 1988: 810, Figs (all misspelled).

*Oxeoschistus euriphile* [sic] Butler; Pyrcz, 2004: 522 (misspelled).

*Oxeoschistus hilara euriphyle* Butler; Lamas, Viloria & Pyrcz, 2004: 211; Chacón & Montero, 2007: 175.

Material examined: COSTA RICA: 1♂: Prov. Cartago, Volcán Turrialba, road from La Pastora, 1900–1950 m, 13.VI.2007, K. Anderson leg., TWP (CEP-MZUJ); 1♂: San José, Santa Eduviges, 1700–2000 m, 02.III.2016, T. Pyrcz leg., CEP-MZUJ; 1♂: Prov. Cartago, Cerro de la Muerte, La Georgina, 2700–2800 m, 9°31′29″N/83°43′7″W, 03.III.2016, T. Pyrcz leg., CEP-MZUJ; 1♂: Prov. San José, Santa Eduviges, 9°29′24″N /83°44′50″W, 1700–1850 m, 04.III.2016, T. Pyrcz leg., CEP-MZUJ; 1♂: Prov. Cartago, Cerro de la Muerte, La Georgina, 2700–2800 m, 9°31′29″N/83°43′7″W, 05.III.2016, T. Pyrcz leg., CEP-MZUJ; 1♂: Prov. San José, Santa Eduviges, 9°29′24″N /83°44′50″W, 1700–1850 m, 08.III.2016, T. Pyrcz leg., CEP-MZUJ; 1♂: Prov. San José, Santa Eduviges, 1700–1850 m, 9°29′24″N /83°44′50″W, 09.III.2016, T. Pyrcz leg., prep. genit. 03_07.08.2017/K.Florczyk, CEP-MZUJ; 1♂: Prov. Cartago, Cerro de la Muerte, La Georgina, 9°32′9″N/83°43′78″, 2800–2900 m, 09.III.2016, T. Pyrcz leg., CEP-MZUJ; 1♂: Prov. San José, Santa Eduviges, 9°29′16″N/83°44′31″W, 1900–2050 m, 10.III.2016, T. Pyrcz leg., CEP-MZUJ; 1♂: Prov. San José, Santa Eduviges, 9°29′16″N/83°44′31″W, 1900–2050 m, 11.III.2016, T. Pyrcz leg., CEP-MZUJ; 1♂: Prov. San José, Santa Eduviges, 1900–2050 m, 9°29′16″N/83°44′31″W, 13.III.2016, T. Pyrcz leg., CEP-MZUJ; 1♀: Prov. San José, Santa Eduviges, 1900–2050 m, 9°29′16″N/83°44′31″W, 11.III.2016, T. Pyrcz leg., prep. Genit. 367_11.04.2016/J. Lorenc, CEP-MZUJ; 1♀: Prov. San José, Santa Eduviges, 1700–1850 m, 09.III.2016, T. Pyrcz leg., CEP-MZUJ. 1♀: Prov. Cartago, Santa Eduviges, 9°29′16″N/83°44′31″W, 1700–2000 m, 02.III.2016, T. Pyrcz leg., prep.genit.02_07.08.2017/K.Florczyk, CEP–MZUJ; 1♂: Prov. San José, Monserrat, above Cascajal de Coronado, 1670 m, 2.VI.2005, I. Nakamura, K. Nishida & A. Damaceno leg., INNY; 1♂: Prov. San José, Reserva Forestal La Trocha, above Cascajal de Coronado, 1700–1740 m, 10°01′17″N/83°55′02″W, 20.IV.2006, I. Nakamura leg., INNY; 1♀: Prov. San José, Bajo La Hondura, 1150–1450 m, 10°03′37″N/83°58′55″W, 13.VI.2005, I. Nakamura leg., INNY; 3♂: Prov. San José, San Gerardo de Rivas to Llano Bonito on trail to Cerro Chirripo, 1870–2500 m, 19.II.2009, I. & M. Nakamura leg., INNY; 1♂: 21.II.2009; 1♂: 22.II.2009; 4♂: 23.II.2009, Prov. San José, Llano Bonito vicinity, on trail to Cerro Chirripo, 2400–2500 m, I. & M. Nakamura & K. Nishida leg., INNY.

#### Remarks

**Fig 7:**
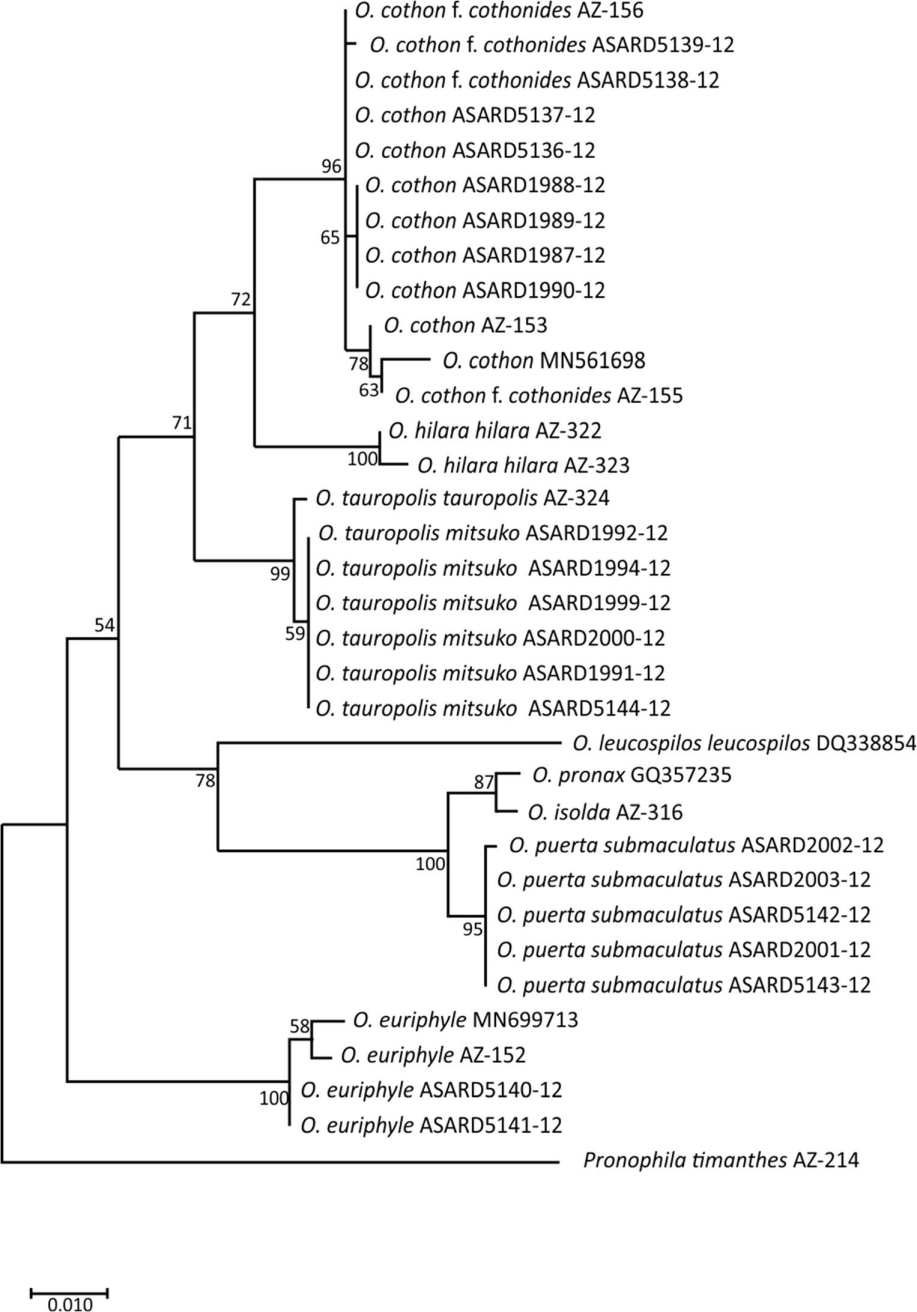
Maximum Likelihood phylogenetic tree of the genus *Oxeoschistus*, based on COI mitochondrial marker with bootstrap branch support values

*Oxeoschistus euriphyle* was described by Butler (1872) as a separate species and has been considered as such by most consulted authors, including DeVries (1987). We do not concur with the systematic arrangement by Lamas *et al* (2004) who consider it a subspecies of *O. hilara* that occurs farther north-west in Honduras, Guatemala and S Mexico. The two have sufficiently different external morphology, including wing colour patterns and differ sufficiently in male genitalia, by the shape of valvae in particular (Fig 5a–c). In *O. euriphyle*, the valvae are elongate, gradually narrowing from base to apex, gently dentate dorsally along apical half, with a somewhat more prominent apical tooth. In *O. hilara*, the valvae are narrower, with a more prominent apical tip and multiple short processes on their dorsal surface. Also, the 8th tergite is considerably broader in *O. hilara* than in *O. euriphyle*. In fact, genitalic differences between *O. hilara* and *O. euriphyle* are more obvious than those between *O. euriphyle* and *O. cothon*. Importantly, preliminary COI data indicate that the two are genetically more distinct from each other than *O. hilara* to *O. cothon* and *O. tauropolis* (Fig 7).

*Oxeoschistus euriphyle* was described from an unspecified locality in Costa Rica. It is restricted to Costa Rica (Talamanca, Meseta Central) and western Panama (Baru). We found it above 1700 and nearly to 3000 m. The altitudinal range for *O. euriphyle* given by DeVries (1987) as 900–2000 m certainly does not correspond to the reality. *O. euriphyle* most frequently occurs at some 2000–2800 m. Above 2500 m, it may be the only representative of the genus. The INBio specimens come from lower (1320–1400 m) and higher elevations (2900 m). The lowest elevation mentioned by DeVries (1987) may have been taken from old specimens’ labels which were frequently inaccurate.

Adults of this species are readily distinguished from any other Costa Rican *Oxeoschistus* and from other Central American congeners by the wide, pale orange transverse band on the HWD extending into FWD where it breaks into a series of oval patches gradually smaller towards subapical area.

### *Oxoeschistus puerta submaculatus* Butler & Druce, 1874 (Figs 4e–h, 5e, 6d)

*Oxeoschistus submaculatus* Butler & Druce, 1874: 339.

*Oxeoschistus submaculatus* Butler & Druce; Godman & Salvin, 1881: 109, pl. 10, Figs 12, 13; Thieme, 1905: 184; d’Abrera, 1988: 808.

*Oxeoschistus puerta* var. *submaculatus* Butler & Druce; Weymer, 1911: 271, pl. 58e; Gaede, 1931: 517.

*Oxeoschistus puerta submaculatus* Butler & Druce; DeVries, 1987: 279, pl. 49, Figs 20, 21; Lamas *et al* 2004: 211; Chacón & Montero, 2007: 176.

Examined material: COSTA RICA: 1♀: Prov. Cartago, Alto Belén above Muńeco, Orosi Valley, 1500–1700 m, 30.VIII.2012, I. Nakamura leg., prep. genit. 1757/06.08.2019 K. Florczyk, CEP-MZUJ; 1♂: Prov. San José, Bajo La Hondura, 1150–1450 m, 10°03′37″N/83°58′55″W, 29.IX.2007, I. Nakamura leg., prep. genit. 1756/06.08.2019 K. Florczyk, CEP-MZUJ; 1♂: Prov. Cartago, Parque Nat. Tapanti, ca.1400 m, 9°44′01″N/83°46′46″W, 18.IX.2004, I. & M. Nakamura leg., INNY; 3♂: Prov. San José, Bajo La Hondura, 1150–1450 m, 10°03′37″N 83°58′55″W, 13.VI.2005, I. Nakamura leg., INNY; 1♀: Prov. Cartago, Alto Belén above Muńeco, Orosi Valley, 1500–1700 m, 9°45′56″N/83°54′04″W, 30.VIII.2008, I. Nakamura leg., INNY; 2♂: 2.XI.2006; 1♂: 9.XI.2006; 1♂: 14.XI.2006, all at Prov. Cartago, Alto Belén above Muńeco, Orosi Valley, 1500–1700 m, I. Nakamura leg., INNY; 1♂: Prov. Heredia, End of Calle Zurqui, 1680 m, 10°03′01″N/84°01′24″W, 24.VIII.2007, I. & M. Nakamura leg., INNY; 1♂: 19.IX.2007; 1♂: 29.IX.2007, Prov. San José, Bajo La Hondura, 10°03′37″N/83°58′55″W, 1150–1450 m, I. Nakamura leg., INNY; 1♂: Prov. Cartago, Guayabo Nat. Monument, 1000 m, 20.IX.2007, I. & M. Nakamura, K. Nishida & R. Alverado leg., INNY; 2♂: Prov. Cartago, Alto Belén above Muńeco, Orosi Valley, 1500–1700 m, 21.VII.2010, I. Nakamura leg., INNY; 1♂: Prov. Limón, Reserva Las Brisas, above Alegria nr. Pocora, 820–1030 m, 10°03′51″N/83°38′04″W, 11.VII.2010, I. Nakamura leg., INNY; 1♀: Prov. Cartago, Alto Belén above Muńeco, Orosi Valley, 1500–1700 m, 30.VIII.2012, I. Nakamura leg., INNY. PANAMA: 1♂: Prov. Darién, Serrania de Pirre, Darién Nat. Park, 1152 m, 7°59′21″N/77°42′26″W, 3.II.2017, I. Nakamura leg., INNY.

#### Remarks

This taxon was originally described as a separate species. However, Weymer (1911) has already treated it as a variation of *Oxeoschistus puerta*. DeVries (1987) and subsequent authors (Lamas *et al*^2004^, Chacón & Montero 2007) also considered it a subspecies of *O. puerta*, a view we concur with, in view of the fact that the differences are basically limited to lighter and wider orange upperside markings of *submaculatus*, whereas undersides and male genitalia (Fig 5e) are indistinguishable from other subspecies (Pyrcz & Viloria 2007, Viloria unpubl.). Female genitalia of other subspecies of *O. puerta* were not studied so far. Those of *O. puerta submaculatus* (Fig 6d) differ from other Central American congeners by the massive paddle-like antevaginal lamellae, the wide ductus bursae, and the ventral position of signa. *O. puerta* is a polytypic species with the nominate subspecies occurring in the Venezuelan Cordillera de La Costa where it is very rare and localized (Viloria *et al*^2010^). An undescribed subspecies occurs in the Perijá range on the Venezuela–Colombia border (Viloria, unpubl.; Pyrcz, unpubl.). It is most probably closely related to *O. isolda*, occurring on the Pacific slopes of the Andes in Colombia, Ecuador and extreme northern Peru (Tumbes), where a population was discovered by IN in the Cerros de Amotape. *O. isolda* differs from *O. puerta* mostly in the considerably darker, brick red, colour of the upperside median bands. The validity of *Oxeoschistus isolda pervius* R. Krüger, 1929, needs to be confirmed since it differs only slightly from the nominate subspecies. Some authors even consider *O. simplex* Butler, *O. duplex* Godman and *O. puerta* conspecific (Lamas *et al*^2004^), which is, in our opinion, incorrect. *O. simplex* and *O. puerta* are locally sympatric in northern Colombia. *O. duplex* cannot be treated as a subspecies of *O. puerta* given, apart from clear colour pattern differences, its geographic distribution (Pyrcz & Rodríguez 2007). DeVries (1987) reports *O. puerta submaculatus* from both slopes of the Cordillera Central (Talamanca and Meseta Central?) within a wide altitudinal band between 800 and 2400 m, and states that it is local but usually common. In South America, *O. puerta* occurs within a very narrow altitudinal band, at some 1400–1800 m, and has never been observed above 2000 m. Interestingly, a population of *O. puerta submaculatus* was recently detected by IN in the Serranía de Pirre Range near the Panama–Colombia border, which is a considerable extension of its range, and a valuable biogeographical record for this mountainous chain situated in an intermediate position between the Andes and Central American mountains.

### Molecular Data

We used 34 sequences of 8 species of *Oxeoschistus* and one species of *Pronophila* to generate a preliminary COI-based phylogenetic tree (Fig 7). Four clades can be distinguished (listed from external branch): the “euriphyle” clade, the “isolda” clade (containing four species), the “tauropolis” clade and the “cothon-hilara” clade. Within the “isolda” clade, the branch containing *O. leucospilos* is placed externally to the branch covering the remaining three species—*O. pronax, O. isolda* and *O. puerta submaculatus*—which is also a branch with the highest support rate within the tree. The average evolutionary divergence over all sequence pairs was 0.05, ranging from 0.03 to 0.11 on intra-specific level and never exceeding 0.01 on subspecific level.

## Discussion

As a result of this study, the number of species of the genus *Oxeoschistus* known from Costa Rica was reduced from five to four, and from Panama to three, because the presence of *O. tauropolis* in Panama is very unlikely in the light of our experience. Nonetheless, the local species diversity of the genus *Oxeoschistus* in Costa Rica surpasses some of the north Andean ranges, such as the Cordillera de Mérida, Sierra de Perijá and the Sierra Nevada de Santa Marta in Colombia where only one species of *Oxeoschistus* is known, and matches that reported from the most biodiverse areas of the Andes in southern Colombia or northern Peru (Pyrcz 2004, Pyrcz & Rodríguez 2007).

Polymorphism affecting phenotypic traits, in particular the colours of the scales and the patterns in butterfly wings, is a common phenomenon. Its evolutionary bases are diverse (Otto & Bourguet 1999, Nijhout 2003). Dimorphism is a particular form of polymorphism in which only two morphs are fixed. Among the Pronophilina, it is a very unusual case, and only documented for some species of the genus *Lymanopoda* Westwood, 1851. In their case, however, there is a predominant form, usually bearing dull markings, and a rare form with conspicuous markings. Such forms are found in *Lymanopoda maletera* Adams & Bernard, 1979, *L. confusa* Brown, 1943 and *L. dietzi* Adams & Bernard, 1981 (Pyrcz *et al* 2009). There are no previously known cases of dimorphic females in the genus *Oxeoschistus* nor in the entire “Pronophila” clade which comprises such related genera as *Pronophila* Doubleday, 1849; *Pseudomaniola* Röber, 1889; *Corades* Hewitson, 1849; *Lasiophila* C. Felder & R. Felder, 1859; and others (Peña *et al*^2006^). Evolutionary backgrounds of this phenomenon in *Oxeoschistus cothon* need to be investigated rigorously. This would seem, however, a difficult endeavour considering the relative rarity of the *cothonides* form in the natural environment. Preliminary observations on the relative frequencies of both forms, and in particular their behaviour, indicate that the form *cothonides* could perhaps be involved in some kind of mimetic relationships with unpalatable models of the genus *Heliconius* Kluk, 1780. The only likely candidate appears to be *Heliconius clysonymus montanus* Salvin, 1871, which has a somewhat similar upperside pattern marked by the forewing oblique yellow band and a large hindwing reddish orange patch. Females of the form *cothonides* were observed to be consistently more sedentary, seen while sunning on low bushes with wings wide open, indeed where also individuals of *Heliconius* were seen. On the other hand, the females of typical form were observed always overflying vigorously bamboo clumps in the same manner as the males.

The species of *Oxeoschistus* occurring in Central America, as suggested by preliminary molecular analysis, belong to three clades, of which two are strictly Central American. The most interesting finding is perhaps that *O. hilara* clusters with *O. cothon* and *O. tauropolis*, whereas *O. euriphyle*, which used to be considered as its conspecific, constitutes a sister-clade to all other examined species including the South American *O. pronax*, *O. isolda* and *O. leucospilos*, suggesting its ancestral position in the genus phylogeny. We need to point out that this is only a preliminary study based on one genetic marker and a more thorough research using longer sequences of DNA is needed to elucidate the relationships among different species and subspecies of *Oxeoschitus* (Pyrcz *et al* in prep.)

**Fig 8:**
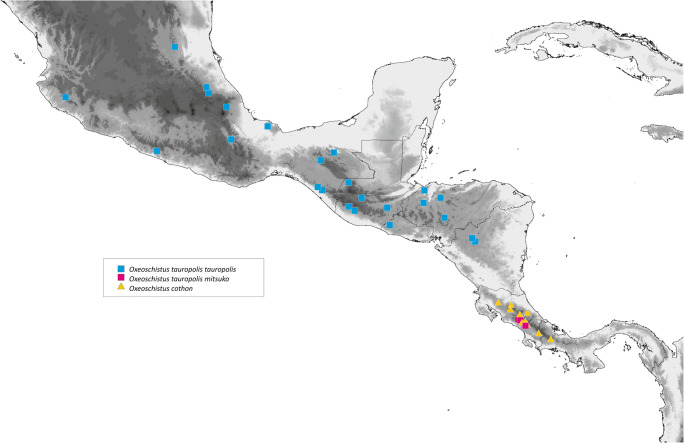
Distribution map of *O. tauropolis* and *O. cothon*

**Fig 9:**
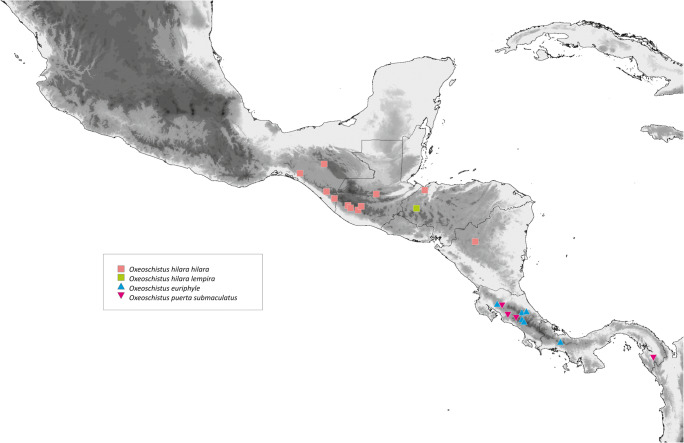
Distribution map of *O. hilara, O. euriphyle* and *O. puerta submaculatus*

*Oxeoschistus puerta submaculatus* Butler & Druce (1874) is the sole Central American representative of a clade, defined by a very high bootstrap support value, widely distributed in the Andes, which includes *O. pronax* (Hewitson, [1850]) and *O. isolda* and, most probably other, morphologically very similar species, *O. simplex* Butler (1868) and *O. duplex* Godman (1905), belong to this group. *O. puerta submaculatus* did not diverge morphologically to any important degree from its Andean allies, and is clearly a product of a recent colonisation from South America through mountain-hopping during one of the colder Pleistocenic intervals. At the maximum of the Wisconsin glaciations, the lower altitudinal limit of cloud forest descended some 500 m below (Rull 2005) current reach, covering the hills extending from northern Colombia through the isthmus of Darién into Panama. *O. puerta submaculatus* has not been found further north of Costa Rica, but its presence in Nicaragua or even Guatemala or Honduras cannot be totally ruled out, given the rarity of this species, the inaccessibility of habitats and its shy nature (Figs 8 and 9).

*Oxeoschistus tauropolis* and *O. cothon* present several similarities in the colour patterns, particularly the unusual large FW and HW yellow median patches, which place them apart from other congeners; thus, not surprisingly, they were considered belonging to a separate genus *Dioriste*. Male genitalia of *O. tauropolis* are however unlike *O. cothon* but also other congeners, particularly the presence of massive valvae with wide dorsal and apical serrate processes. Despite their colour pattern similarities, other morphological traits as well as molecular data indicate that the two species diverged at an early stage of the radiation of *Oxeoschistus*. It is impossible at this time, due to the lack of a robust, calibrated molecular phylogeny of the genus *Oxeoschistus* to infer its origins either in the Andes or Central America. However, it is worth pointing out that the *Oxeoschistus* fauna of Central American mountains harbours four possible paleonedemic species, alongside other exclusively Central American elements, the genus *Drucina* with two species, *Drucina leonata* and *Drucina championi* Godman & Salvin, 1881, and *Arhuaco dryadina* (Schaus, 1913). They are the key species to be investigated from the perspective of historical biogeography of the subtribe Pronophilina.

## Electronic supplementary material

ESM Tables (PDF 94.2 kb)

ESM Tables (PDF 164 kb)
