# Supplementary material for: Revisional Notes on the Cloud Forest Butterfly Genus Oxeoschistus Butler in Central America (Lepidoptera: Nymphalidae: Satyrinae)
Source: Neotrop Entomol. 2020 Mar 14;49(3):392–411. doi: 10.1007/s13744-019-00757-7 (PMC7253525; doi:10.1007/s13744-019-00757-7)
Supplement: Supplementary file 1 — (PDF 94.2 kb) [file 13744_2019_757_MOESM1_ESM.pdf]

Accession	Genus	species	subspecies	Country	Region	Locality	Altitude [m]	Specimen in	Sequence source
**MN699713**	*Oxeoschistus*	*euriphyle*		Costa Rica	San Jose	Cerro de la Muerte, Division Sta Eduviges	1900-2050	CEPUJ	Pyrcz et al., 2018
**MN699714**	*Oxeoschistus*	*euriphyle*		Costa Rica	San Jose	Cerro de la Muerte, Division Sta Eduviges	1900-2050	CEPUJ	this study (AZ-152)
**MN709596**	*Oxeoschistus*	*cothon*		Costa Rica	San Jose	Cerro de la Muerte, Division Sta Eduviges	1900-2050	CEPUJ	this study (AZ-153)
**MN561698**	*Oxeoschistus*	*cothon*		Costa Rica	San Jose	Cerro de la Muerte, Division Sta Eduviges	1900-2050	CEPUJ	Pyrcz et al., 2018
**MN709595**	*Oxeoschistus*	*cothon*	f. *cothonides*	Costa Rica	San Jose	Cerro de la Muerte, Division Sta Eduviges	1900-2050	CEPUJ	this study (AZ-155)
**MN709594**	*Oxeoschistus*	*cothon*	f. *cothonides*	Costa Rica	San Jose	Cerro de la Muerte, Division Sta Eduviges	1900-2050	CEPUJ	this study (AZ-156)
**MN709590**	*Oxeoschistus*	*isolda*		Costa Rica	San Jose	Cerro de la Muerte, Division Sta Eduviges	1900-2050	CEPUJ	this study (AZ-316)
**MN709593**	*Oxeoschistus*	*hilara*	*hilara*	Guatemala	Quetzalten	Zunil, Las Georginas	2400-2450	CEPUJ	this study (AZ-322)
**MN709592**	*Oxeoschistus*	*hilara*	*hilara*	Guatemala	Chilasco	Chilasco	1800	CEPUJ	this study (AZ-323)
**MN709591**	*Oxeoschistus*	*tauropolis*	*tauropolis*	Guatemala	Suchitepec	Res. Los Terrales, Vesubio- Atitlas	1000-1050	CEPUJ	this study (AZ-324)
**MN709589**	*Pronophila*	*timanthes*		Costa Rica	San Jose	Cerro de la Muerte, Division- sta. Eduviges	1900-2050	CEPUJ	this study (AZ-214)
**ASARD1987-12**	*Oxeoschistus*	*cothon*		Costa Rica	Cartago	La Union, San Rafael, La Carpintera, Campo Escuela Istaru	1750	INB-CR	BOLD Systems
**ASARD1988-12**	*Oxeoschistus*	*cothon*		Costa Rica	Cartago	La Union, San Rafael, La Carpintera, Campo Escuela Istaru	1750	INB-CR	BOLD Systems
**ASARD1989-12**	*Oxeoschistus*	*cothon*		Costa Rica	Cartago	La Union, San Rafael, La Carpintera, Campo Escuela Istaru	1750	INB-CR	BOLD Systems
**ASARD1990-12**	*Oxeoschistus*	*cothon*		Costa Rica	Cartago	La Union, San Rafael, La Carpintera, Campo Escuela Istaru	1750	INB-CR	BOLD Systems
**ASARD1991-12**	*Oxeoschistus*	*tauropolis*	*mitsuko*	Costa Rica	Heredia	Santo Domingo, Santa Rosa, IN Bioparque	1100	INB-CR	BOLD Systems
**ASARD1992-12**	*Oxeoschistus*	*tauropolis*	*mitsuko*	Costa Rica	Heredia	Santo Domingo, Santa Rosa, IN Bioparque	1100	INB-CR	BOLD Systems
**ASARD1994-12**	*Oxeoschistus*	*tauropolis*	*mitsuko*	Costa Rica	Heredia	Santo Domingo, Santa Rosa, IN Bioparque	1100	INB-CR	BOLD Systems
**ASARD1999-12**	*Oxeoschistus*	*tauropolis*	*mitsuko*	Costa Rica	Heredia	Santo Domingo, Santa Rosa, IN Bioparque	1100	INB-CR	BOLD Systems
**ASARD2000-12**	*Oxeoschistus*	*tauropolis*	*mitsuko*	Costa Rica	Heredia	Santo Domingo, Santa Rosa, IN Bioparque	1100	INB-CR	BOLD Systems
**ASARD2001-12**	*Oxeoschistus*	*puerta*	*submaculatus*	Costa Rica	Alajuela	Guatuso, Buenavista, Estacion Pilon, 1,5 km SO C. Carmela	800	INB-CR	BOLD Systems
**ASARD2002-12**	*Oxeoschistus*	*puerta*	*submaculatus*	Costa Rica	Cartago	Jimenez, Pejilaye, Estacion Biologica Copal	1040	INB-CR	BOLD Systems
**ASARD2003-12**	*Oxeoschistus*	*puerta*	*submaculatus*	Costa Rica	Limon	Talamanca, Bratsi, Sendero entra Laguna Dabagri y Laguna Sacabico	1080	INB-CR	BOLD Systems
**ASARD5136-12**	*Oxeoschistus*	*cothon*		Costa Rica	Cartago	Area de conservacion La Amistad Pacifico, Paraiso, Send. Oropendola 3	1250	INB-CR	BOLD Systems
**ASARD5137-12**	*Oxeoschistus*	*cothon*		Costa Rica	Cartago	Area de conservacion La Amistad Pacifico, Paraiso, Send. Pava Catarata 2	1350	INB-CR	BOLD Systems
**ASARD5138-12**	*Oxeoschistus*	*cothon*	f. *cothonides*	Costa Rica	Cartago	Area de conservacion La Amistad Pacifico, Paraiso, Send. Pava Catarata 1	1350	INB-CR	BOLD Systems
**ASARD5139-12**	*Oxeoschistus*	*cothon*	f. *cothonides*	Costa Rica	Cartago	Area de conservacion La Amistad Pacifico, Paraiso, Send. Pava Catarata 5	1350	INB-CR	BOLD Systems
**ASARD5140-12**	*Oxeoschistus*	*euriphyle*		Costa Rica	Cartago	Area de conservacion La Amistad Pacifico, Paraiso, Send. Pava Catarata 3	1350	INB-CR	BOLD Systems
**ASARD5141-12**	*Oxeoschistus*	*euriphyle*		Costa Rica	Cartago	Area de conservacion La Amistad Pacifico, Paraiso, Send. Pava Catarata 1	1350	INB-CR	BOLD Systems
**ASARD5142-12**	*Oxeoschistus*	*puerta*	*submaculatus*	Costa Rica	Alajuela	Area de conservacion Arenal, San Ramon, Villa Blanca 3	1050	INB-CR	BOLD Systems
**ASARD5143-12**	*Oxeoschistus*	*puerta*	*submaculatus*	Costa Rica	Alajuela	Area de conservacion Arenal, San Ramon, Villa Blanca 4	1050	INB-CR	BOLD Systems
**ASARD5144-12**	*Oxeoschistus*	*tauropolis*	*mitsuko*	Costa Rica	Alajuela	Area de conservacion Arenal, San Ramon, Villa Blanca 2	1050	INB-CR	BOLD Systems
**DQ338854**	*Oxeoschistus*	*leucospilos*	*leucospilos*	Peru	Junin	no data	no data	no data	GenBank
**GQ357235**	*Oxeoschistus*	*pronax*				no data	no data	no data	GenBank
