# Supplementary material for: Revisional Notes on the Cloud Forest Butterfly Genus Oxeoschistus Butler in Central America (Lepidoptera: Nymphalidae: Satyrinae)
Source: Neotrop Entomol. 2020 Mar 14;49(3):392–411. doi: 10.1007/s13744-019-00757-7 (PMC7253525; doi:10.1007/s13744-019-00757-7)
Supplement: Supplementary file 2 — (PDF 164 kb) [file 13744_2019_757_MOESM2_ESM.pdf]

		1	2	3	4	5	6	7	8	9	10	11	12	13	14	15	16	17	18	19	20	21	22	23	24	25	26	27	28	29	30	31	32	33
O. cothon ASARD1987-12	**1**																																	
O. cothon ASARD1988-12	**2**	0,00																																
O. cothon ASARD1990-12	**3**	0,00	0,00																															
O. cothon ASARD1989-12	**4**	0,00	0,00	0,00																														
O. cothon ASARD5137-12	**5**	0,00	0,00	0,00	0,00																													
O. cothon ASARD5136-12	**6**	0,00	0,00	0,00	0,00	0,00																												
O. cothon AZ-153	**7**	0,00	0,00	0,00	0,00	0,00	0,00																											
O. cothon MN561698	**8**	0,01	0,01	0,01	0,01	0,01	0,01	0,01																										
O. cothon f. *cothonides* AZ-155	**9**	0,01	0,01	0,01	0,01	0,01	0,01	0,00	0,01																									
O. cothon f. *cothonides* AZ-156	**10**	0,00	0,00	0,00	0,00	0,00	0,00	0,00	0,01	0,00																								
O. cothon f. *cothonides* ASARD5138-12	**11**	0,00	0,00	0,00	0,00	0,00	0,00	0,00	0,01	0,01	0,00																							
O. cothon f. *cothonides* ASARD5139-12	**12**	0,00	0,00	0,00	0,00	0,00	0,00	0,00	0,01	0,01	0,00	0,00																						
O. hilara hilara AZ-322	**13**	0,03	0,03	0,03	0,03	0,03	0,03	0,03	0,04	0,03	0,03	0,03	0,03																					
O. hilara hilara AZ-323	**14**	0,03	0,03	0,03	0,03	0,03	0,03	0,03	0,04	0,03	0,03	0,03	0,03	0,00																				
O. tauropolis tauropolis AZ-324	**15**	0,03	0,03	0,03	0,03	0,03	0,03	0,03	0,04	0,03	0,03	0,03	0,03	0,03	0,04																			
O. tauropolis mitsuko ASARD1991-12	**16**	0,03	0,03	0,03	0,03	0,03	0,03	0,03	0,04	0,03	0,03	0,03	0,03	0,03	0,04	0,00																		
O. tauropolis mitsuko ASARD1992-12	**17**	0,03	0,03	0,03	0,03	0,03	0,03	0,03	0,04	0,03	0,03	0,03	0,03	0,03	0,04	0,00	0,00																	
O. tauropolis mitsuko ASARD1994-12	**18**	0,03	0,03	0,03	0,03	0,03	0,03	0,03	0,04	0,03	0,03	0,03	0,03	0,03	0,04	0,00	0,00	0,00																
O. tauropolis mitsuko ASARD1999-12	**19**	0,03	0,03	0,03	0,03	0,03	0,03	0,03	0,04	0,03	0,03	0,03	0,03	0,03	0,04	0,00	0,00	0,00	0,00															
O. tauropolis mitsuko ASARD2000-12	**20**	0,03	0,03	0,03	0,03	0,03	0,03	0,03	0,04	0,03	0,03	0,03	0,03	0,03	0,04	0,00	0,00	0,00	0,00	0,00														
O. tauropolis mitsuko ASARD5144-12	**21**	0,03	0,03	0,03	0,03	0,03	0,03	0,03	0,04	0,03	0,03	0,03	0,03	0,03	0,04	0,00	0,00	0,00	0,00	0,00	0,00													
O. leucospilos leucospilos DQ338854	**22**	0,06	0,06	0,06	0,06	0,06	0,06	0,07	0,08	0,07	0,06	0,06	0,06	0,07	0,07	0,06	0,06	0,06	0,06	0,06	0,06	0,06												
O. pronax GQ357235	**23**	0,06	0,06	0,06	0,06	0,06	0,06	0,06	0,07	0,07	0,06	0,06	0,06	0,07	0,07	0,07	0,06	0,06	0,06	0,06	0,06	0,06	0,07											
O. isolda AZ-316	**24**	0,06	0,06	0,06	0,06	0,06	0,06	0,06	0,07	0,07	0,06	0,06	0,06	0,07	0,07	0,06	0,06	0,06	0,06	0,06	0,06	0,06	0,07	0,01										
O. puerta submaculatus ASARD2001-12	**25**	0,06	0,06	0,06	0,06	0,06	0,06	0,06	0,07	0,07	0,06	0,06	0,06	0,06	0,07	0,06	0,06	0,06	0,06	0,06	0,06	0,06	0,07	0,01	0,01									
O. puerta submaculatus ASARD2002-12	**26**	0,06	0,06	0,06	0,06	0,06	0,06	0,06	0,07	0,07	0,06	0,06	0,06	0,07	0,07	0,06	0,06	0,06	0,06	0,06	0,06	0,06	0,07	0,01	0,01	0,00								
O. puerta submaculatus ASARD2003-12	**27**	0,06	0,06	0,06	0,06	0,06	0,06	0,06	0,07	0,07	0,06	0,06	0,06	0,06	0,07	0,06	0,06	0,06	0,06	0,06	0,06	0,06	0,07	0,01	0,01	0,00	0,00							
O. puerta submaculatus ASARD5142-12	**28**	0,06	0,06	0,06	0,06	0,06	0,06	0,06	0,07	0,07	0,06	0,06	0,06	0,06	0,07	0,06	0,06	0,06	0,06	0,06	0,06	0,06	0,07	0,01	0,01	0,00	0,00	0,00						
O. puerta submaculatus ASARD5143-12	**29**	0,06	0,06	0,06	0,06	0,06	0,06	0,06	0,07	0,07	0,06	0,06	0,06	0,06	0,07	0,06	0,06	0,06	0,06	0,06	0,06	0,06	0,07	0,01	0,01	0,00	0,00	0,00	0,00					
O. euriphyle ASARD5140-12	**30**	0,05	0,05	0,05	0,05	0,05	0,05	0,05	0,06	0,06	0,05	0,05	0,05	0,06	0,06	0,05	0,05	0,05	0,05	0,05	0,05	0,05	0,07	0,07	0,07	0,06	0,06	0,06	0,06	0,06				
O. euriphyle ASARD5141-12	**31**	0,05	0,05	0,05	0,05	0,05	0,05	0,05	0,06	0,06	0,05	0,05	0,05	0,06	0,06	0,05	0,05	0,05	0,05	0,05	0,05	0,05	0,07	0,07	0,07	0,06	0,06	0,06	0,06	0,06	0,00			
O. euriphyle MN699713	**32**	0,06	0,06	0,06	0,06	0,06	0,06	0,06	0,06	0,06	0,06	0,06	0,06	0,06	0,06	0,06	0,05	0,05	0,05	0,05	0,05	0,05	0,08	0,07	0,07	0,06	0,07	0,06	0,06	0,06	0,01	0,01		
O. euriyphyle AZ-152	**33**	0,06	0,06	0,06	0,06	0,05	0,05	0,05	0,06	0,06	0,05	0,05	0,06	0,06	0,06	0,05	0,05	0,05	0,05	0,05	0,05	0,05	0,07	0,07	0,07	0,06	0,06	0,06	0,06	0,06	0,01	0,01	0,01	
Pronophila timanthes AZ-214	**34**	0,09	0,09	0,09	0,09	0,09	0,09	0,09	0,10	0,10	0,09	0,09	0,09	0,09	0,10	0,08	0,09	0,09	0,09	0,09	0,09	0,09	0,10	0,11	0,10	0,10	0,10	0,10	0,10	0,10	0,09	0,09	0,10	0,09
